# RAFT polymers for protein recognition

**DOI:** 10.3762/bjoc.6.66

**Published:** 2010-06-17

**Authors:** Alan F Tominey, Julia Liese, Sun Wei, Klaus Kowski, Thomas Schrader, Arno Kraft

**Affiliations:** 1 Chemistry, School of Engineering & Physical Sciences, Heriot-Watt University, Riccarton, Edinburgh EH14 4AS, United Kingdom; 2 Universität Duisburg-Essen, Fachbereich Chemie, Institut für Organische Chemie, Universitätsstraße 5, 45117 Essen, Germany

**Keywords:** electrostatic interactions, hydrophobic effect, isothermal calorimetry, protein recognition, RAFT polymers

## Abstract

A new family of linear polymers with pronounced affinity for arginine- and lysine-rich proteins has been created. To this end, *N*-isopropylacrylamide (NIPAM) was copolymerized in water with a binding monomer and a hydrophobic comonomer using a living radical polymerization (RAFT). The resulting copolymers were water-soluble and displayed narrow polydispersities. They formed tight complexes with basic proteins depending on the nature and amount of the binding monomer as well as on the choice of the added hydrophobic comonomer.

## Introduction

The ability of biological receptors to bind strongly and specifically to a particular molecular target is an essential part of biological machinery. The best example is the immune system where antibodies are generated in response to minute amounts of foreign antigens. A continual challenge in nanoscale chemistry is to mimic the biological molecular recognition functions by synthetic chemistry with the aim of producing systems of lower complexity. When successful, this will enable the manufacturing of robust and specific synthetic receptors for a given protein target [1]. Proteins are a formidable challenge in this respect because they represent large macromolecules with a characteristic shape, size and highly complex functionalized surface. Artificial protein receptors are desired for protein enrichment and purification, sensing and diagnostics applications, as well as therapeutic uses involving interference with critical protein–protein interactions.

Multivalency represents the key to generate high-affinity materials for biomacromolecules with a sufficient number of binding sites for Coulomb attraction and hydrophobic interactions [2]. A statistical evaluation of crystal structures led to the discovery that hot spots in protein–protein contact areas are enriched in aromatic amino acids and in arginine. These are often surrounded by energetically less important residues that most likely serve to occlude bulk solvent from the hot spot and lower the local dielectric constant [3–4].

With this principle in mind, several groups have designed relatively simple linear polymeric structures with branched ionic comonomers and thus achieved remarkable affinities and biological properties. In their elegant work, Kulkarni et al. reported the use of NIPAM-based copolymers for lysozyme recovery by affinity thermoprecipitation. These polymers contained multiple acetamido groups in a hydrophilic environment for maximum interaction with the catalytic cleft and achieved high affinities [5]. Rotello and Thayumanavan have described amphiphilic polymer scaffolds, which nonspecifically bound to chymotrypsin, inhibited its peptidase activity and modulated substrate specificity; very high ionic strengths again released the protein from the polymer [6–7].

Protein recognition by multifunctional polymeric hosts features two prominent advantages. On one hand, it simplifies the complex recognition interface to isolated 1:1 complexes between monomeric binding sites and single complementary amino acid residues, while simultaneously allowing for an extensive induced-fit process of the linear polymer on the protein surface – in other words they encourage polymer/protein self-assembly in order to maximize attractive noncovalent interactions.

A second major advantage of multivalent polymeric hosts is their rapid and efficient synthesis at low cost as well as the high proteolytic stabilities of most polymer backbones. They also pose fewer racemization problems which often accompany proteinogenic amino acids in peptidic environments.

In recent years, our group has developed water-soluble linear polymeric protein binders which contained one or more different binding monomers and displayed micromolar protein affinities [8], accompanied in a number of cases with promising protein selectivities [9]. These linear polymers were all prepared by free radical copolymerization in DMF followed by deprotection of the binding monomers in polymer-analogous transformations. Thus, a polymerized bisphosphonate tetramethyl ester was subjected to LiBr-assisted nucleophilic cleavage to furnish the free bisphosphonate dianion binding site. This procedure has two major drawbacks. First, if the functional groups on the polymer backbone become restricted in their accessability, the final deprotection step will suffer from low conversion rates. Second, the resulting material is polydisperse, rendering the characterization of the protein binding event problematic. Even with incorporated fluorescence labels, the overall emission intensity change resulting from protein addition will reflect only a virtual averaged value, because short and long chains will bind simultaneously, most likely with different affinities and stoichiometries. A quantitative description must inherently suffer from this averaging effect.

## Results and Discussion

Reversible addition–fragmentation chain transfer (RAFT) polymerization [10] and atom-transfer radical polymerization (ATRP) have become extremely useful tools for the controlled synthesis of a wide range of polymers and could solve both problems by formation of monodisperse functionalized polymer chains of equal length, without the need for final polymer-analogous deprotection. So far, there have been no reports of the successful use of ATRP with acrylamides. In contrast, RAFT can be used in a variety of solvents and, most importantly, it is compatible with NIPAM [11–12]. For this reason, RAFT was chosen in this paper as the preferred method for controlled synthesis of linear polymers.

**Figure 1:**
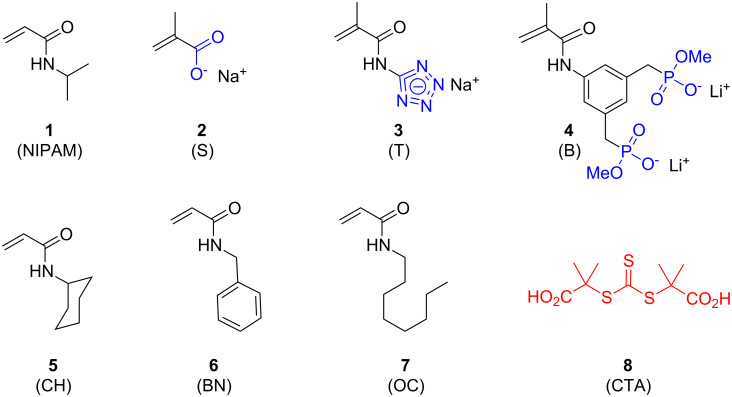
Structures of monomers **1**–**7** and chain transfer agent **8** used in the RAFT polymerizations.

For initial screenings we selected a combination of anionic and hydrophobic binding monomers (Figure 1) that were well suited for simultaneous recognition of basic amino acids (Lys/Arg) as well as nonpolar residues (Val, Leu, Ile, Phe). NIPAM was chosen as the main comonomer because it forms polymers which are water-soluble at room temperature and even allow thermoprecipitation with a bound protein guest. NIPAM-based polymers are also reminiscent of peptides since both contain an amide group in the repeat unit. RAFT makes use of a chain transfer agent (CTA) for which we selected the water-soluble trithiocarbonate **8** [13–14] which efficiently caps the growing polymer chain, but can be completely removed from the final polymer by reaction with an excess of AIBN and selective polymer precipitation into hexane [11].

Three anionic comonomers suitable for binding lysine and arginine were chosen from earlier work with linear polymers and microgels [9,15–16]: Sodium methacrylate (**2**) (S), polymerizable tetrazolate **3** (T) and bisphosphonate **4** (B). These anionic comonomers were directly copolymerized with NIPAM and a hydrophobic acrylamide. The latter carried cyclohexyl (CH), benzyl (BN) or octyl (OC) moieties as hydrophobic residues. In the polymer designation code, the first letter indicates the anionic comonomer used (S, T or B), the subsequent number its mol % in the monomer mixture; the two-letter abbreviation (CH, BN or OC) stands for the hydrophobic comonomer used, again followed by the mol %; the balance to 100 mol % was made up by NIPAM. For example, S10CH10 means that this RAFT copolymer was made from sodium methacrylate (10 mol %), *N*-cyclohexylacrylamide (10 mol %), and NIPAM (80 mol %).

RAFT polymerizations were carried out in methanol at 60 °C for 48 hours in the presence of CTA **8** and azo initiator V-50. The monomer concentration was 0.75 M, the molar ratio of [V-50]/[CTA] was 3, and the concentration of CTA and V-50 were adjusted to target polymers with a molecular weight of 3000, 7000 or 17000 g mol ^−1^ at full conversion. This is possible since the degree of polymerization under RAFT conditions is equal to the ratio between monomer and chain transfer reagent concentration. Conversion was almost 100%, and copolymers were isolated by precipitation in hexane. The absence of low-molecular weight impurities such as monomers was ascertained by ^1^H NMR spectroscopy. Molecular weights were determined by gel-permeation chromatography (GPC) analysis of the copolymers. Narrow polydispersities (≤1.3) were observed for the shorter copolymers, although the highest molecular weights (targeted at 17000 g mol^−1^) reached only experimental values of 11,000–12,000 g mol^−1^ and also produced slightly higher polydispersities (1.56). For comparison, some copolymers such as S20CH15 were also prepared with a molecular weight of ~3000 g mol^−1^.

**Figure 2:**
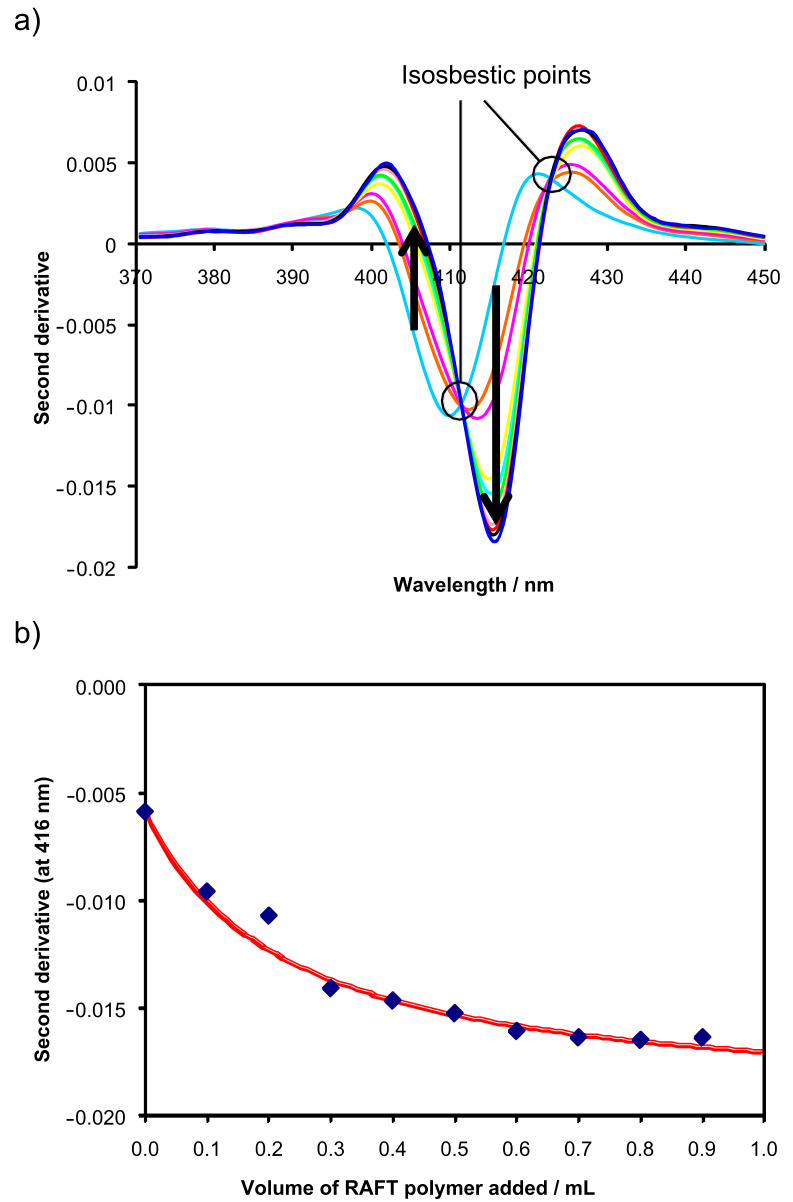
a) Second derivative UV–vis spectra [17–19] observed during a full titration of a stock solution of RAFT copolymer S10CH15 (6.3 × 10^−3^ mol L^−1^) into a solution of cyt C (9.9 × 10^−6^ mol L^−1^) in phosphate buffer at pH 7 and ionic strength of 0.15 mol L^−1^ KCl. The arrows indicate increasing amounts of RAFT copolymer added. b) Plot of second derivative values at 416 nm as a function of volume (in mL) of RAFT copolymer solution added. The filled diamonds are experimental values, whereas the drawn curve represents the calculated isotherm for a *K*_a_ of 1.6 × 10^3^ M^−1^ assuming 1:1 binding [20].

Titrations were first carried out by UV–vis spectroscopy with cytochrome C, a protein carrying a chromophore. Second derivative spectra were calculated using the Savitzky–Golay algorithm [17–19]. The second derivative is a useful method of refining the spectra to reveal subtle changes in the UV–vis absorption plot. The UV titration of a typical RAFT copolymer into a solution of cytochrome C in a phosphate buffer (pH 7, 0.15 M KCl) showed characteristic second derivative spectra, similar to those observed in the titrations of microgels into protein solutions [16]. Isosbestic points are clearly visible along with a bathochromic shift of the absorbance peak (Figure 2a). A dissociation constant of 1.6 × 10^3^ M^−1^ could be fitted to the binding isotherm when the second derivative values of the protein at 415 nm were plotted against the RAFT polymer concentration (Figure 2b). Cytochrome C already showed noticable and selective binding to microgels [16] containing 10 mol % sodium methacrylate and RAFT copolymers of similar composition. Unlike microgels whose molecular weight is very high (typically 10^6^–10^8^ g mol^−1^), cytochrome C possesses a relatively small molecular weight similar to the RAFT copolymers. As a result, the RAFT copolymers and cytochrome C favor 1:1 binding. The incorporation of a hydrophobic comonomer further improved binding. The maximum binding strength was observed for polymers containing 15 mol % of *N*-cyclohexylacrylamide and 20 mol % of sodium methacrylate (Table 1).

**Table 1:** UV–vis titrations of cytochrome C with selected RAFT copolymers.

RAFT Copolymer^a^	Macroscopic *K*_a_ / M^−1^	Polymer : Protein Stoichiometry

**S10CH10**	400	1:1
**S10BN10**	n.d.	n.d.
**S10OC10**	20	1:1
**S10CH15**	1600	1:1
**S20CH15**	>2000	n.d.

^a^S = sodium methacrylate, CH = *N*-cyclohexylacrylamide, BN = *N*-benzylacrylamide, OC = *N*-octylacrylamide.

**Figure 3:**
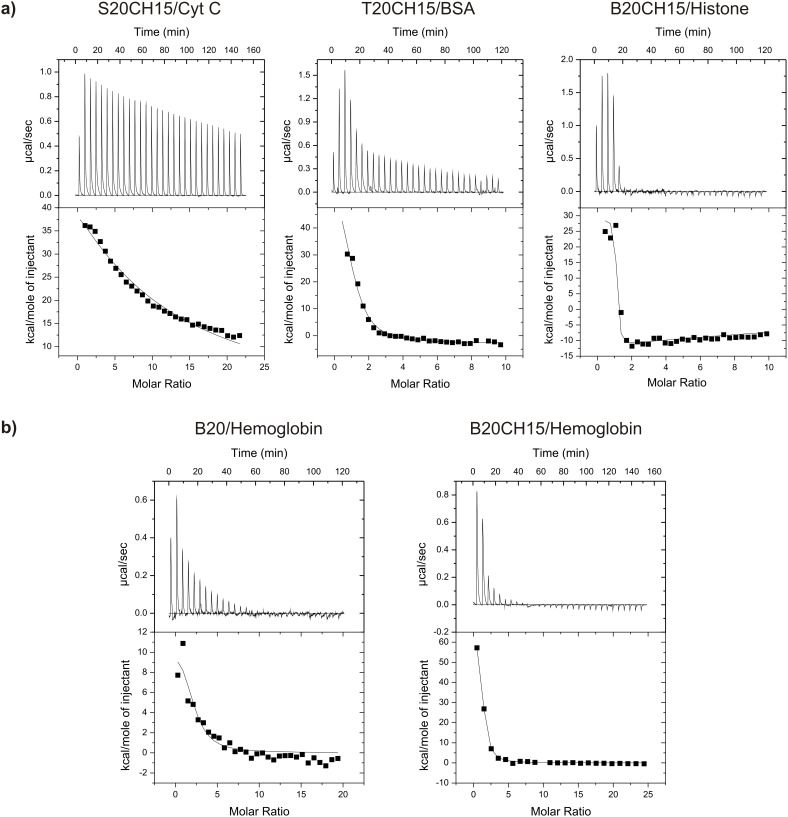
Isothermal calorimetric binding curves for selected polymer/protein host–guest pairs. a) Typical binding curves with representative proteins for the major polymers based on the three anionic binding sites. Note the marked affinity increase from sodium methacrylate over tetrazolate to bisphosphonate dianion. b) Binding curves of two bisphosphonate RAFT copolymers, one without and one with the hydrophobic *N*-cyclohexylacrylamide comonomer (15 mol %). The contribution of the nonpolar cyclohexyl monomer towards hemoglobin binding is evident from the steeper slope of the binding curve.

For an independent comparison, the same protein–polymer pairs were subsequently subjected to microcalorimetric titrations (Figure 3), which confirmed the major trends gained from spectroscopic detection but differed in several details (Table 2). Specifically, RAFT copolymers S10CH10, S10BN10, S10OC10, S10CH15 and S20CH15 were examined in their complex formation with cytochrome C (MW 14 kD, pI 9.2) and hemoglobin (MW 68 kD, pI 7.0). Negligible heat changes were observed for all titrations with sodium methacrylate-containing polymers, consistent with the small *K*_a_ values already determined by UV–vis titrations (20–1600 M^−1^); obviously, the methacrylate anion is a weak binder for lysines and arginines on these protein surfaces. Moderate binding (3 × 10^4^ M^−1^) was only detected with S20CH15, which carries twice the amount of carboxylate groups. Association constants were initially calculated for each 1:1 complexation event of a single protein by the copolymer [20]. However, even with S20CH15, no binding was detectable with hemoglobin, confirming an interesting cytochrome C preference of all sodium methacrylate-carrying polymers, which also corresponded to previous results with microgels [16].

By contrast, tetrazolate copolymer T20CH15 and bisphosphonate copolymer B20CH15 showed large enthalpy changes and hence much higher *K*_a_ values (>10^6^ M^−1^) which were about two orders of magnitude higher than those achieved with sodium methacrylate copolymer S20CH15 (~10^4^ M^−1^). This is not surprising for the bisphosphonate, which carries twice the amount of negative charges. However, the monoanionic tetrazolate anion is very similar in acidity and hydrogen bond pattern to a carboxylate, so that similar affinities would have been expected. Most likely, the difference is explained by interactions with the π-face of the tetrazolate anion, which are not possible with a carboxylate.

In all cases, protein complexation by RAFT polymers was endothermic, i.e., entropy-driven. Hence, unspecific electrostatic attraction in combination with solvophobic forces contributed the most towards protein binding.

To quantify the contribution of nonpolar comonomers, hemoglobin was also titrated with pure tetrazolate and bisphosphonate copolymers. Intriguingly, *K*_a_ values dropped substantially by 1–2 orders of magnitude (see Table 1: T20 vs T20CH15). In other words, the random incorporation of cyclohexyl comonomers into the polymer was beneficial for the protein recognition event. Close inspection of thermodynamic data revealed that the entropy term was responsible for this increased affinity. We therefore tentatively explain the gain in free energy by an increased classical hydrophobic effect due to the presence of additional nonpolar cyclohexyl residues throughout the polymer chain.

**Figure 4:**
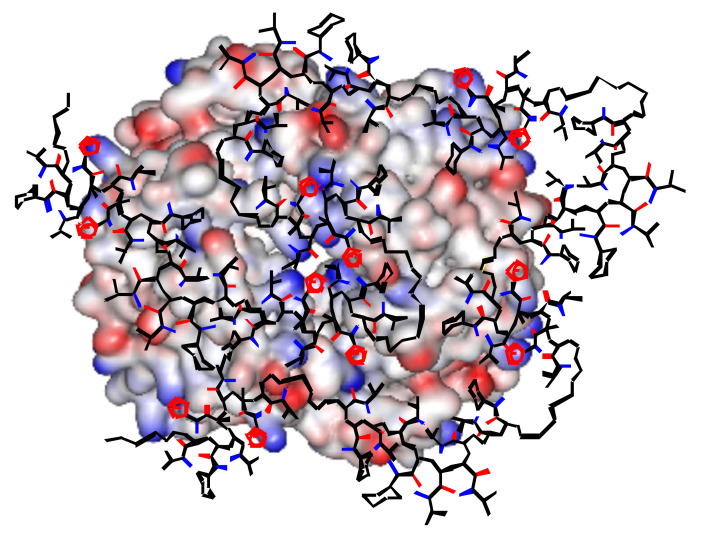
Graphical illustration of the potential binding mode on hemoglobin tetramer (represented as electrostatic potential surface, lysines = blue). The RAFT copolymer T20CH15 (tetrazole rings = red) undergoes an extensive induced fit procedure on the protein surface maximizing unspecific electrostatic and hydrophobic contacts. Some NIPAM sidechains were omitted for clarity.

For biological applications, it is desirable to keep the polymer size close to the size of the protein, so that specific 1:1 complexation is favored (Figure 4). In order to investigate this assumption, the sodium methacrylate polymer S20CH15 was titrated as a short oligomer (MW 3000 g mol^−1^) and an average-size polymer (MW 12000 g mol^−1^). Direct comparison produced a drastic difference: No binding could be detected for the short version, indicating that size matters and promotes multivalent or cooperative binding.

Finally, the protein series was extended to lysine-rich histone (pI 10), lysozyme (pI 9), proteinase K (pI 8) and bovine serum albumin or BSA (pI 6). Again, the strong binders B20CH15 and T20CH15 were examined concerning their affinities towards proteins of varying pI (Table 2). In direct comparison, the bisphosphonate seems to be superior to the tetrazolate. While B20CH15 stayed well below micromolar *K*_d_ values even with BSA, T20CH15 hardly ever reached the micromolar regime. Obviously, the bisphosphonate’s high negative charge density is especially effective for protein surfaces with a high density of basic amino acids such as the DNA-binding histones or for those offering distinct clusters of cationic amino acid residues (e.g. BSA). Interestingly, although in most cases nonlinear regression converges with an assumed 1:1 complex stoichiometry, curve fitting is greatly improved with a sequential binding or 2-sites model [21]. In all these cases, the first polymer binds very tightly to the protein surface, but leaves significant room for a second polymer forming an – admittedly much weaker – 2:1 complex. Histone association with B20CH15 is an illustrative example. The first *K*_d_ value is 16 nM, followed by very weak binding at a second site with a *K*_d_ of 1 mM. With respect to varying pI values, both RAFT polymers display little selectivity: From lysozyme (pI > 9) down to BSA (pI < 6) protein affinities vary by less than one order of magnitude.

**Table 2:** Microcalorimetric protein titrations with RAFT polymers.

RAFT copolymer^a^	Protein^b^	Macroscopic *K*_a _/ M^−1^	Polymer : protein	*K*_a_ per residue / M^–1^	Monomer : protein	*ΔG* / kcal mol^−1^	*ΔH* / kcal mol^−1^	*TΔS* / kcal mol^−1^

**S10CH10**	Cyt C	NA	–	–	–	–	–	–
**S10BN10**	Cyt C	NA	–	–	–	–	–	–
**S10OC10**	Cyt C	NA	–	–	–	–	–	–
**S10CH15**	Cyt C	NA	–	–	–	–	–	
**S10CH10**	Hem	NA	–	–	–	–	–	–
**S20CH15**^b^	Cyt C	NA	–	–	–	–	–	–
**S20CH15**^c^	Cyt C	3 × 10^4^	7:1	9 × 10^2^	15:1	–	–	–
**S20CH15**^c^	Hem	NA	–	–	–	–	–	–
**T20**	Hem	~2 × 10^4^	–	~9 × 10^3^	–	–	–	–
**T20CH15**	His	8 × 10^5^ → 5 × 10^3^	2 sites	2 × 10^4^	–	–	–	–
	Lys	8 × 10^5^ → 5 × 10^3^	2 sites	1 × 10^4^	7:1	−5.5	+21.2	+26.7
	Prot K	4 × 10^5^ → 3 × 10^3^	2 sites	3 × 10^3^	13:1	−4.6	+17.7	+22.3
	Hem	4 × 10^6^	3:1	1 × 10^4^	78:1	−5.7	+4.2	+9.9
	BSA	4 × 10^5^ → 3 × 10^3^	10:1	6 × 10^3^	6:1	−5.2	+4.4	+9.6
**B20**	Hem	7 × 10^5^	2:1	7 × 10^4^	20:1	−6.6	+1.2	+7.8
**B20CH15**	His	6 × 10^7^ → 7 × 10^2^	2 sites	2 × 10^5^	18:1	−7.4	+2.4	+9.8
	Lys	1 × 10^6^ → 3 × 10^3^	2:1	4 × 10^4^	15:1	−6.3	+0.7	7.0
	Prot K	NA	–	–	–	–	–	–
	Hem	4 × 10^6^	1:1	2 × 10^5^	15:1	−7.2	+5.1	+12.3
	BSA	2 × 10^6^	3:1	9 × 10^4^	5:1	−6.7	+15.4	+22.1

^a^S = sodium methacrylate, T = tetrazolate **3**, B = bisphosphonate **4**, CH = *N*-cyclohexylacrylamide, BN = *N*-benzylacrylamide, OC = *N*-octylacrylamide. ^b^Cyt C = cytochrome C; Hem = hemoglobin; His = histone; Lys = lysozyme; Prot K = proteinase K; BSA = bovine serum albumin. ^c^MW ~3000 g mol^−1^. ^d^MW ~17000 g mol^−1^. NA indicates that no binding constant and thermodynamic data were obtained from microcalorimetry titrations, because heat changes were too small.

## Conclusion

In summary, RAFT copolymerization of NIPAM with monomers containing anionic binding sites for basic amino acids led to polymers of low polydispersities which were effective protein binders in buffered aqueous solution, with tunable stoichiometries close to the ideal 1:1 ratio. Although molecular recognition is based on unspecific electrostatic attraction and hydrophobic forces, those proteins which feature a high density of positive charges on their surfaces are bound especially well by the bisphosphonate site, in some cases reaching micromolar or sub-micromolar *K*_d_ values. Copolymerization with *N*-cyclohexylacrylamide introduced additional nonpolar groups beneficial for protein binding, leading to a substantial entropy gain and significantly improving protein affinities. The best pair was a bisphosphonate-containing RAFT copolymer and lysine–rich histone (*K*_d_ = 16 nM). In the future, we intend to investigate if it is possible to interrupt the nucleosome complex formation by noncovalent detachment of ds-DNA from its “own” histone proteins using histone-binding RAFT copolymers.

## Supporting Information

File 1 Full experimental procedures, characterization details, microcalorimetry measurements, UV titration procedures and potentiometric titrations.
