# Supplementary material for: RAFT polymers for protein recognition
Source: Beilstein J Org Chem. 2010 Jun 17;6:66. doi: 10.3762/bjoc.6.66 (PMC2919265; doi:10.3762/bjoc.6.66)
Supplement: File 1 — Full experimental procedures, characterization details, microcalorimetry measurements, UV titration procedures and potentiometric titrations. [file Beilstein_J_Org_Chem-06-66-s001.pdf]

Supporting Information

for

RAFT polymers for protein recognition

Alan F. Tominey[1], Julia Liese[1], Sun Wei[2], Klaus Kowski[2], Thomas Schrader[*2] and Arno Kraft[*1]

Address: [1]Chemistry, School of Engineering & Physical Sciences, Heriot-Watt University, Riccarton, Edinburgh EH14 4AS, United Kingdom and [2]Universität Duisburg-Essen, Fachbereich Chemie, Institut für Organische Chemie, Universitätsstraße 5, 45117 Essen, Germany

Email: Arno Kraft - A.Kraft@hw.ac.uk; Thomas Schrader - Thomas.Schrader@uni-due.de

[*]Corresponding author

Full experimental procedures, characterization details, microcalorimetry measurements, UV titration procedures and potentiometric titrations.

General

All chemicals used were reagent-grade and used without further purification. Polymerizable bisphosphonate **4** was prepared as described in the literature [1]. The water and methanol-soluble azo initiator V-50, or 2,2'-azobis(2-methylpropionamidine) dihydrochloride, has a half-life of 10 h at 56 °C and was purchased from Sigma-Aldrich. ^{1}H NMR spectra were obtained with a Bruker AC200 spectrometer (200 MHz) or Bruker DPX400 spectrometer (400 MHz). Differential scanning calorimetry (DSC) analyses were performed with a Thermal Advantage DSC 2010 at a heating rate of 20 °C min^{-1} under a constant nitrogen flow. UV–vis spectra were recorded with a Shimadzu UV-1601 UV–vis spectrophotometer.

N-Cyclohexyl acrylamide (5)

Cyclohexylamine (25.8 g, 0.26 mol) and triethylamine (26.8 g, 0.26 mol) were dissolved in CH_2Cl_2 (50 mL) with ice cooling and vigorous stirring. A solution of acryloyl chloride (20.0 g, 0.22 mol) in CH_2Cl_2 was added dropwise over a period of 1 h. The solution was left to warm to room temperature and stirred overnight. The solvent was evaporated under vacuum to give a pale yellow waxy powder. The residue was then recrystallized from hexane to provide off-white crystals. Yield: 31.6 g (94%). ^{1}H NMR (200 MHz, DMSO-d_6): δ 1.16 (m, 5H, -CH_2-), 1.63 (m, 5H, -CH_2-), 2.50 (m, 1H, CH), 5.49 (dd, 1H, CH, J = 2.6, 12.3 Hz), 6.14 (m, 2H, CH_2), 8.03 (d, 1H, NH, J = 7.7 Hz). DSC: T_m 112 °C (Lit. mp. 112–113 °C).[2]

N-Benzyl acrylamide (6)

Benzylamine (27.9 g, 0.26 mol) and triethylamine (26.8 g, 0.26 mol) were dissolved in CH_2Cl_2 (50 mL) with ice cooling and vigorous stirring. A solution of acryloyl chloride (20.0 g, 0.22 mol) in CH_2Cl_2 was added dropwise over a period of 1 h. The solution was left to warm to room temperature and stirred overnight. The solvent was evaporated under vacuum to afford a pale yellow, waxy powder. The residue was then recrystallized from hexane to provide off-white crystals. Yield: 30.6 g (86%). ^{1}H NMR (200 MHz, DMSO-d_6): δ 4.35 (d, 2H, CH_2, J = 6.0 Hz), 5.53 (dd, 1H, CH, J = 2.5, 9.9 Hz), 6.14 (m, 2H, CH_2), 7.25 (m, 5H, C_6H_5), 8.73 (t, 1H, NH, J = 4.8 Hz). DSC T_m 63 °C (Lit. m.p. 65–65.7 °C).[3]

N-Octyl acrylamide (7)

Octylamine (34.2 g, 0.26 mol) and triethylamine (26.8 g, 0.26 mol) were dissolved in CH_2Cl_2 (50 mL) with ice cooling and vigorous stirring. A solution of acryloyl chloride (20.0 g, 0.22 mol) in CH_2Cl_2 was added dropwise over a period of 1 h. The solution was left to warm to room temperature and stirred overnight. The solvent was evaporated under vacuum to yield a pale yellow, waxy powder. The residue was then recrystallized from hexane to provide off-white crystals. Yield: 38.4 g (95%). ^{1}H NMR (200 MHz, DMSO-d_6): δ 0.84 (t, 3H, CH_3, J = 6.7 Hz), 1.4 [m, 12H, $(CH_2)_6$], 3.95 (q, 2H, CH_2, J = 6.6 Hz), 5.53 (dd, 1H, =CH, J = 2.8, 9.6 Hz), 6.14 (m, 2H, =CH_2), 8.07 (t, 1H, NH, J = 6.6 Hz). DSC: T_m 34.5 °C (Lit. m.p. 35–36 °C).[4]

S,S'-Bis(α,α'-dimethyl-α''-acetic acid)-trithiocarbonate (8)[5,6]

Carbon disulfide (6.85 g, 90.0 mmol), acetone (12.1 g, 0.23 mol), chloroform (26.9 g, 0.23 mol) and decyltrimethylammonium bromide (0.50 g, 1.78 mol) were mixed with hexane (30 mL) in a 3-necked round-bottomed flask under nitrogen. The solution was cooled to ~10 °C in

an ice-water bath. Sodium hydroxide solution (50%, 50.4 g, 0.63 mol) was added dropwise over 60 min while the temperature was kept below 25 °C. The solution was then stirred overnight. Water (200 mL) was added, followed by concentrated HCl (30 mL) with vigorous stirring and nitrogen purging for 30 min. The yellow solid was filtered, washed with water and dried under vacuum. Yield: 2.66 g (21%). ^{1}H NMR (200 MHz, DMSO-d_6): δ 1.59 (s, 12 H). ^{13}C NMR (50 MHz, CD_3OD): δ 25.65 (4 × CH_3), 57.14 (2 × CMe_2), 176.13 (2 × COOH), 220.29 (C═S). MS (EI): *m*/*z* 282 (M^+), 162, 87, 45.

Typical synthesis of RAFT copolymers

S10: NIPAM (1.488 g, 13.1 mmol) and sodium methacrylate (0.142 g, 1.3 mmol) were dissolved in methanol (20 mL) in a polymerization tube fitted with a septum cap (Figure 1). The mixture was degassed by bubbling nitrogen through it for 30 min. *S*,*S*'-Bis(α,α'-dimethyl-α''-acetic acid)-trithiocarbonate (**8**) (74.4 mg, 0.26 mmol) and azo initiator V-50 (23.8 mg, 0.08 mmol) were then added and the mixture was heated to 60 °C for 48 h. The solvent was evaporated, the residue dissolved in THF and precipitated into pentane, then left to dry to give a pale yellow, odorous powder. ^{1}H NMR (200 MHz, D_2O): δ 1.02 (bs, CH_3), 1.14 (br. s, 9H, CH_3), 1.47 (br. t, CH_2), 1.96 (br. m, CH), 3.84 (br. s, CH), 7.53–7.88 (br. m, NH). IR (KBr, cm^{-1}): 3300, 1650, 1500, 1400, 1225, 1200, 1050.

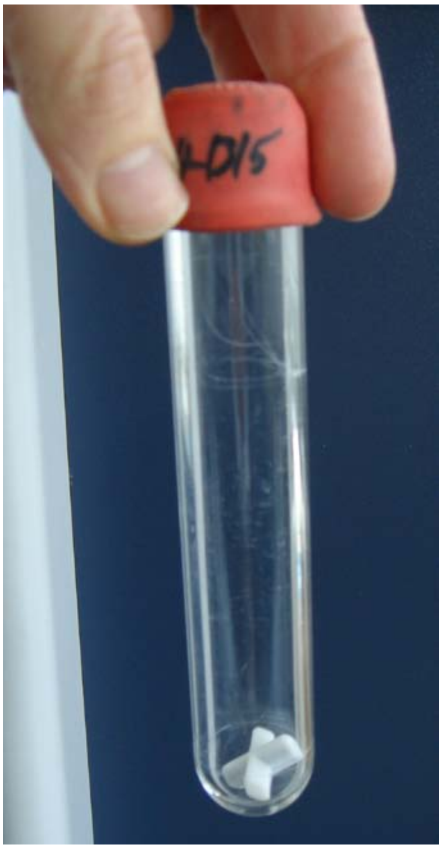

Figure 1: A polymerization tube consisting of a thick-walled test-tube with a septum cap, was used in the preparation of the RAFT copolymers.

S10CH15: NIPAM (1.488 g, 13.1 mmol), sodium methacrylate (0.142 g, 1.3 mmol) and *N*-cyclohexylacrylamide (0.209 g, 1.3 mmol) was dissolved in methanol (20 mL) in a polymerisation tube fitted with a septum cap. The mixture was degassed by bubbling nitrogen through it for 30 min. *S*,*S*'-Bis(α,α'-dimethyl-α''-acetic acid)-trithiocarbonate (**8**) (74.4 mg,

0.26 mmol) and azo initiator V-50 (23.8 mg, 0.08 mmol) were then added and the mixture was heated to 60 °C for 48 h. The solvent was evaporated, the residue dissolved in THF and precipitated into pentane, then left to dry to give a pale yellow, odorous powder. ^{1}H NMR (200 MHz, D_2O): δ 1.07 (br. s, CH_3), 1.37 (br. m, 9H, CH_3), 1.50–1.90 (br. m, CH + CH_2), 3.80 (br. s, CH). IR (KBr, cm^{-1}): 3300, 1650, 1500, 1400, 1225, 1200, 1050.

GPC

GPC measurements were carried out with Polymer Laboratories PL-gel mixed-B columns (10 mm particle size, 100 – 10^6 Å pore size, effective MW range 10^3 – 10^7 g mol^{-1}, column length 3×30 cm, fitted with guard columns) and a refractive index detector. Samples were injected through a Rheodyne 7725i injection port with a 200 μL loop. The column was calibrated with a series of poly(ethylene glycol)/poly(ethylene oxide) standards using aqueous sodium nitrate (0.2 M)/sodium hydrogen orthophosphate (0.1 M) as eluent. Samples were made up at concentrations of approx. 2.0 mg mL^{-1} and filtered prior to injection. Elution time for GPC runs was 30 min. A typical analysis is shown in Figure 2.

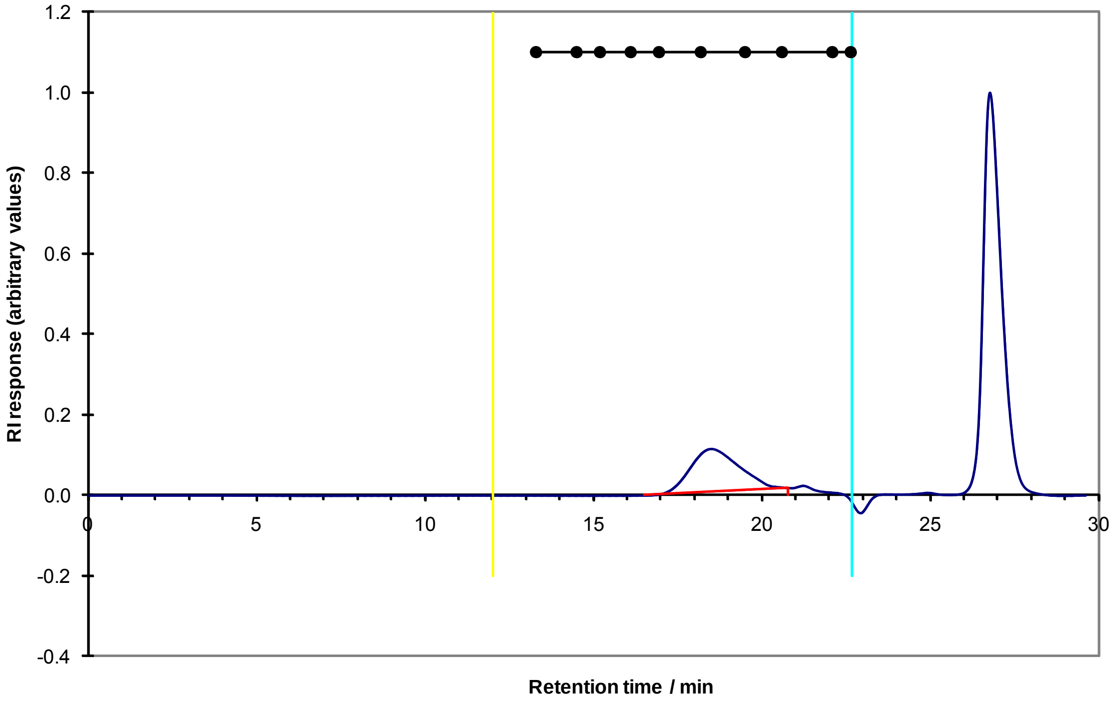

Figure 2: GPC trace for RAFT copolymer S10CH15: M_n 3800 g mol^{-1}, M_w 4900 g mol^{-1}, polydispersity index M_w/M_n 1.31.

UV–vis binding studies

The pH 7.0 phosphate buffer with an ionic strength of 0.15 mol L^{-1} was made up by dissolving NaH_2PO_4 (0.920 g, 7.67 mmol), Na_2HPO_4 (0.567 g, 3.99 mmol) and KCl (9.848 g, 0.132 mol) in 1 L of de-ionized water.

A stock solution of protein was prepared in pH 7.0 phosphate buffer; a typical concentration of a protein stock solution was 1.02×10^{-5} mol L^{-1} for cytochrome C. A solution of 20 mg of

polymer in 0.5 mL of protein stock solution was used as the sample solution for the titration. A solution of microgel in buffer solution (20 mg in 0.5 mL) served as the blank solution.

A reference cell was filled with 2.5 mL of phosphate buffer in a quartz cuvette. The sample cuvette was filled with 2.5 mL of protein stock solution. Equal aliquots of sample solution and blank solution were titrated into the sample cuvette and the blank cell, respectively, to maintain the same concentration of polymer in each cuvette. UV–vis spectra were recorded after the solution had equilibrated during 15 min. of stirring. Titrations were carried out at room temperature (20 °C).

The UV–vis absorbance vs wavelength data was pasted into a spreadsheet and the second derivative was calculated according to the Savitsky–Golay[7-9] method with the help of a macro [9]. Since the second derivative of the absorbance is insensitive to light scattering, this gave a clearer representation of the changes in the UV–vis spectrum of the protein that occurred during binding of the protein to the RAFT copolymer.

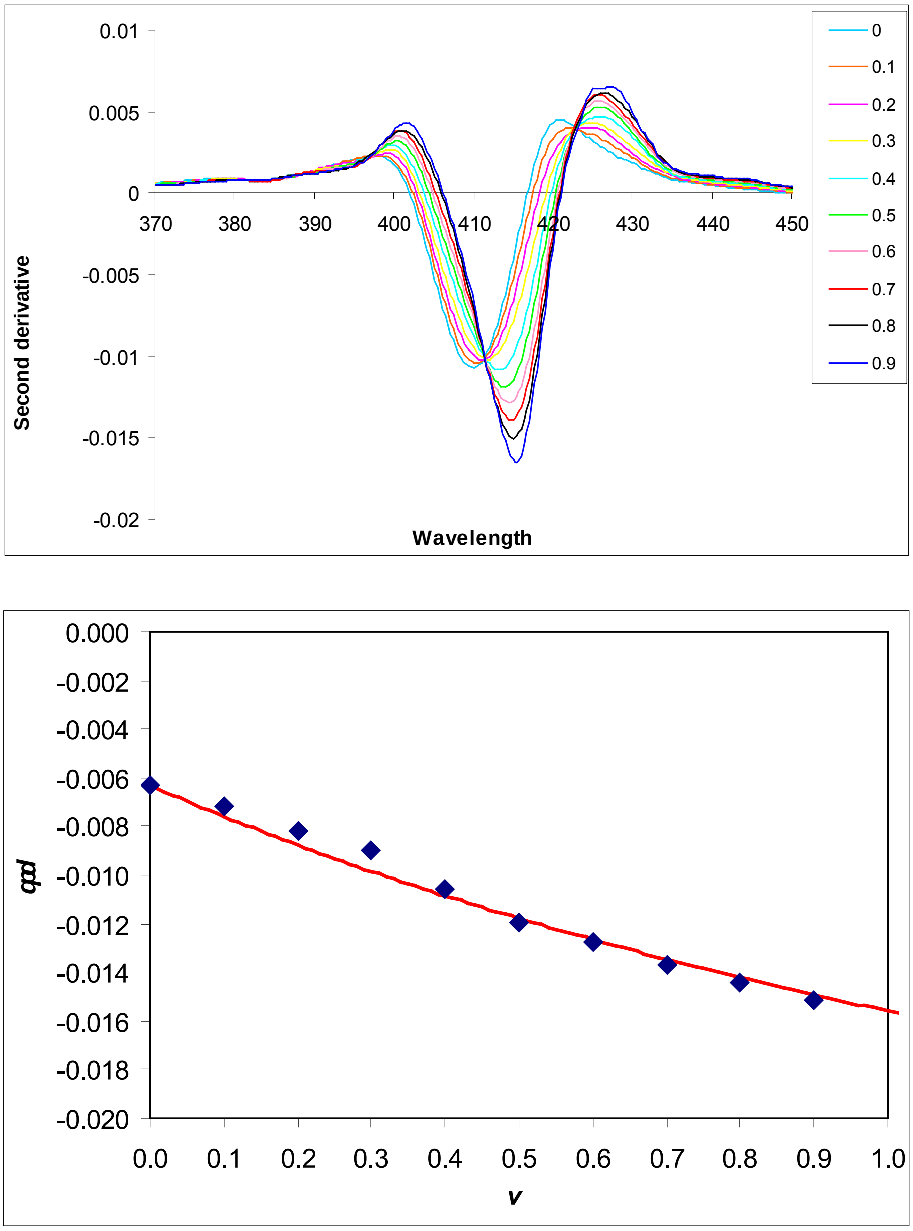

Figure 3: Top: second-derivative UV–vis spectra observed during a titration of a stock solution of S10OC10 into a solution of cytochrome C. Below: corresponding isotherm for complex formation between S10OC10 and cytochrome C; the drawn curve represents the calculated isotherms for 1:1 binding, whereas the filled diamonds are experimental values.

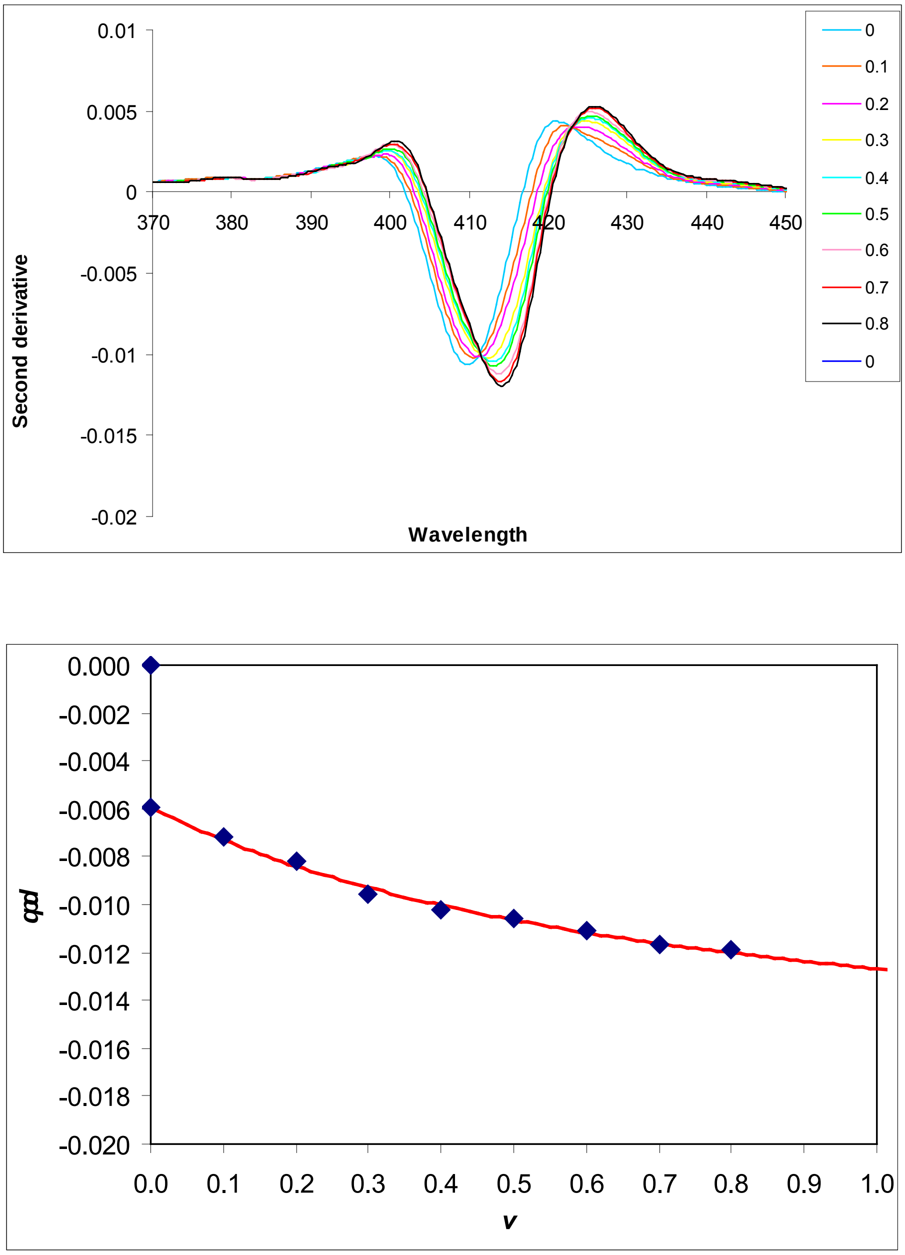

Figure 4: Top: second-derivative UV–vis spectra observed during a titration of a stock solution of S10CH10 into a solution of cytochrome C. Below: isotherm for complex formation between S10CH10 and cytochrome C; the drawn curve represents the calculated isotherms for 1:1 binding, whereas the filled diamonds are experimental values.

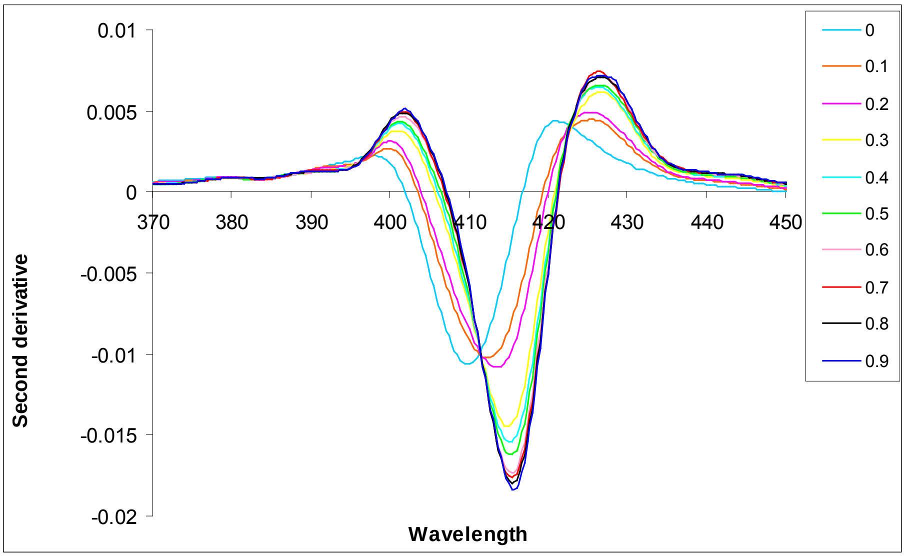

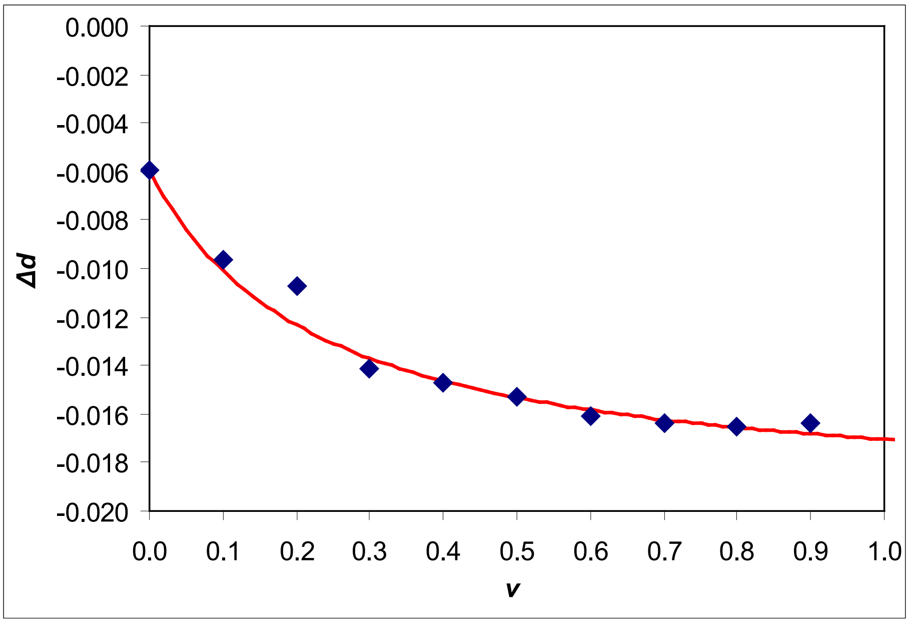

Figure 5: Top: second-derivative UV–vis spectra observed during a titration of a stock solution of S10CH15 into a solution of cytochrome C. Below: corresponding isotherm for complex formation between S10CH15 and cytochrome C; the drawn curve represents the calculated isotherms for 1:1 binding, whereas the filled diamonds are experimental values.

ITC titration

ITC titrations were performed with micro-calorimeter VP-ITC at 25 °C. The initial volume of the solution in the cell was 1.4211 mL. 10 mM Hepes (pH 7) was used as the buffer. In all the titrations, protein solutions were used as hosts in the cell and polymer solutions as ligands. The dilution effect of the polymers were measured as reference and subtracted from the titrations before the calculation. Two kinds of evaluation methods incorporated in the software were used to get the best fittings and smallest errors: one set of sites and sequential binding with 2 binding sites. The macroscopic binding constant, change of enthalpy and entropy are given here. For every pair, two titration curves are shown. The left curve is calculated with the concentration of the polymer, and the right one is calculated with the concentration of the functional monomer.

1. B20CH15 vs. Histone

Sequential binding two sites

$K_1 = 5.93E7 \pm 11\%\ M^{-1}$ $\Delta H_1 = 28.7 \pm 7\%$ kcal M^{-1} $\Delta S_1 = 0.130$ kcal $M^{-1}\ K^{-1}$

$K_2 = 774 \pm 10\%\ M^{-1}$ $\Delta H_2 = -3.87E3 \pm 94\%$ kcal M^{-1} $\Delta S_2 = -13.0$ kcal $M^{-1}\ K^{-1}$

Table S1: ITC titration data between B20CH15 and Histone in buffer.

$C_{Histone}$ [M]	$C_{B20CH15}$ [M]	Ratio $C_{B20CH15}/C_{Histone}$	Heat exchange [µcal]
3.89E-06	1.76E-06	0.45	55.54
3.86E-06	2.93E-06	0.76	53.45
3.83E-06	4.09E-06	1.07	60.95
3.81E-06	5.24E-06	1.38	15.79
3.78E-06	6.38E-06	1.69	0.14
3.75E-06	7.51E-06	2.00	-2.56
3.73E-06	8.63E-06	2.31	0.09
3.70E-06	9.75E-06	2.64	-0.81
3.67E-06	1.09E-05	2.96	-1.77
3.65E-06	1.20E-05	3.27	0.29
3.62E-06	1.31E-05	3.60	0.81
3.60E-06	1.41E-05	3.93	-3.03
3.57E-06	1.52E-05	4.26	-2.88
3.55E-06	1.63E-05	4.58	-2.09
3.52E-06	1.73E-05	4.92	-2.26
3.50E-06	1.84E-05	5.25	-2.6
3.47E-06	1.94E-05	5.59	-0.3
3.45E-06	2.05E-05	5.93	-2.65
3.42E-06	2.15E-05	6.28	-2.31
3.40E-06	2.25E-05	6.61	-2.67
3.38E-06	2.35E-05	6.95	-2.74
3.35E-06	2.45E-05	7.31	-1.13
3.33E-06	2.55E-05	7.65	-2.67
3.30E-06	2.65E-05	8.02	-2.94
3.28E-06	2.74E-05	8.37	-3.17
3.26E-06	2.84E-05	8.71	-3.88
3.23E-06	2.94E-05	9.09	-3.55
3.21E-06	3.03E-05	9.44	-3.15
3.19E-06	3.13E-05	9.80	-1.92

Dilution effect of B20CH15 in buffer

Added volume μL	$C_{B20CH15}$ [M]	Heat exchange [μcal]
5	3.65E-07	1.46
10	1.09E-06	2.13
10	1.81E-06	1.84
10	2.53E-06	1.09
10	3.24E-06	1.68
10	3.95E-06	1.03
10	4.65E-06	0.57
10	5.34E-06	0.55
10	6.03E-06	0.25
10	6.72E-06	-0.18
10	7.40E-06	-0.32
10	8.08E-06	-0.21
10	8.75E-06	-0.48
10	9.41E-06	-0.50
10	1.01E-05	-0.32
10	1.07E-05	-0.54
10	1.14E-05	-0.65
10	1.20E-05	-0.80
10	1.27E-05	-0.65
10	1.33E-05	-0.83
10	1.39E-05	-0.66
10	1.45E-05	-0.78
10	1.52E-05	-0.61
10	1.58E-05	-0.70
10	1.64E-05	-0.93
10	1.70E-05	-0.72
10	1.76E-05	-0.77
10	1.82E-05	-0.91
10	1.88E-05	-0.87
10	1.94E-05	-0.95

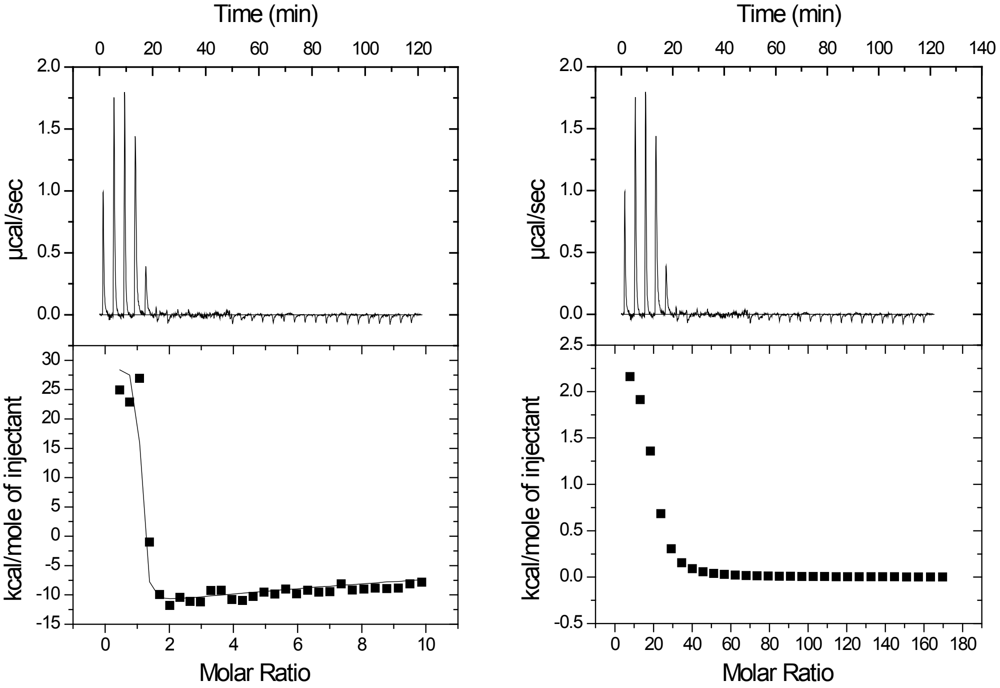
Time (min)
µcal/sec
kcal/mole of injectant
Molar Ratio
Time (min)
µcal/sec
kcal/mole of injectant
Molar Ratio

2. B20CH15 vs. Lysozyme

Sequential binding two sites

K_1 = 1.09E6±60% M^{-1} ΔH_1 = 14.9±24% kcal M^{-1} ΔS_1 = 0.077 kcal M^{-1} K^{-1}

K_2 = 3.45E03±32% M^{-1} ΔH_2 = −2.16±27% kcal M^{-1} ΔS_2 = −0.071 kcal M^{-1} K^{-1}

Table S2: ITC titration data between B20CH15 and Lysozyme in buffer.

$C_{Lysozyme}$ [M]	$C_{B20CH15}$ [M]	Ratio $C_{B20CH15}/C_{Lysozyme}$	Heat exchange [µcal]
3.44E-06	5.71E-07	0.17	7.06
3.41E-06	1.71E-06	0.50	26.07
3.39E-06	2.84E-06	0.84	5.99
3.37E-06	3.96E-06	1.18	4.18
3.34E-06	5.07E-06	1.52	3.24
3.32E-06	6.18E-06	1.86	1.73
3.30E-06	7.27E-06	2.20	0.22
3.27E-06	8.36E-06	2.56	-0.38
3.25E-06	9.44E-06	2.90	-1.76
3.23E-06	1.05E-05	3.25	-1.73
3.20E-06	1.16E-05	3.62	-2.30
3.18E-06	1.26E-05	3.97	-2.17
3.16E-06	1.37E-05	4.33	-1.81
3.14E-06	1.47E-05	4.69	-2.26
3.12E-06	1.58E-05	5.05	-3.24
3.09E-06	1.68E-05	5.43	-2.90
3.07E-06	1.78E-05	5.79	-2.95
3.05E-06	1.88E-05	6.16	-2.53
3.03E-06	1.98E-05	6.53	-3.11
3.01E-06	2.08E-05	6.91	-2.67
2.99E-06	2.18E-05	7.28	1.49
2.96E-06	2.28E-05	7.69	1.59
2.94E-06	2.37E-05	8.07	1.75
2.92E-06	2.47E-05	8.45	1.55
2.90E-06	2.56E-05	8.84	1.78
2.88E-06	2.66E-05	9.23	1.40
2.86E-06	2.75E-05	9.62	0.36
2.84E-06	2.84E-05	10.01	0.29
2.82E-06	2.94E-05	10.41	1.79
2.80E-06	3.03E-05	10.81	1.66

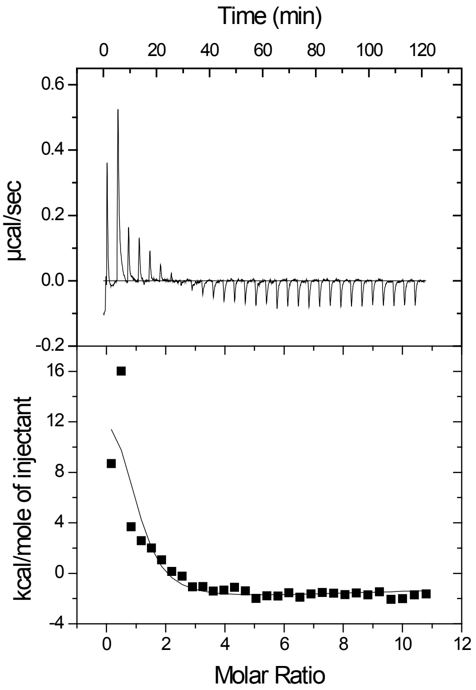

Time (min)
µcal/sec
kcal/mole of injectant
Molar Ratio

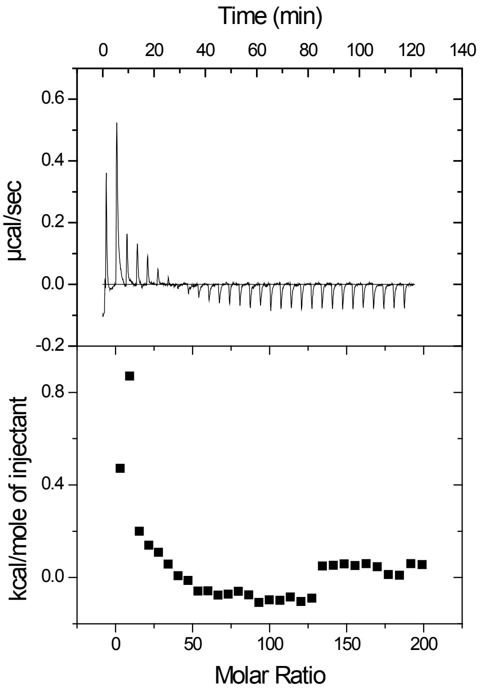

Time (min)
µcal/sec
kcal/mole of injectant
Molar Ratio

3. B20CH15 vs. BSA

N = 5.29 ± 45%   K = 2.65E6 ± 28% M^{-1}
ΔH = 15.4 ± 49% kcal M^{-1} ΔS = 0.074 kcal M^{-1} K^{-1}

Table S3: ITC titration data between B20CH15 and BSA in buffer.

C_{BSA} [M]	$C_{B20CH15}$ [M]	Ratio $C_{B20CH15}/C_{BSA}$	Heat exchange [µcal]
1.37E-06	5.05E-07	0.37	55.30
1.36E-06	1.51E-06	1.11	47.04
1.35E-06	2.51E-06	1.86	17.99
1.34E-06	3.50E-06	2.61	11.32
1.33E-06	4.48E-06	3.37	6.76
1.32E-06	5.46E-06	4.14	5.22
1.31E-06	6.43E-06	4.91	2.81
1.3E-06	7.39E-06	5.68	1.50
1.29E-06	8.34E-06	6.47	1.11
1.28E-06	9.29E-06	7.26	-0.65
1.27E-06	1.02E-05	8.06	-1.01
1.26E-06	1.12E-05	8.87	-1.32
1.25E-06	1.21E-05	9.67	-0.93
1.25E-06	1.30E-05	10.41	-1.70
1.24E-06	1.39E-05	11.23	-2.65
1.23E-06	1.48E-05	12.06	-2.09
1.22E-06	1.57E-05	12.89	-1.93
1.21E-06	1.66E-05	13.74	-2.16
1.2E-06	1.75E-05	14.58	-1.90
1.19E-06	1.84E-05	15.45	-1.91
1.19E-06	1.93E-05	16.18	-2.20
1.18E-06	2.01E-05	17.04	-2.21
1.17E-06	2.10E-05	17.92	-2.58
1.16E-06	2.18E-05	18.80	-2.50
1.15E-06	2.27E-05	19.70	-2.38
1.14E-06	2.35E-05	20.61	-2.84
1.14E-06	2.43E-05	21.32	-2.09
1.13E-06	2.51E-05	22.24	-2.98
1.12E-06	2.60E-05	23.17	-2.46
1.11E-06	2.68E-05	24.10	-2.41

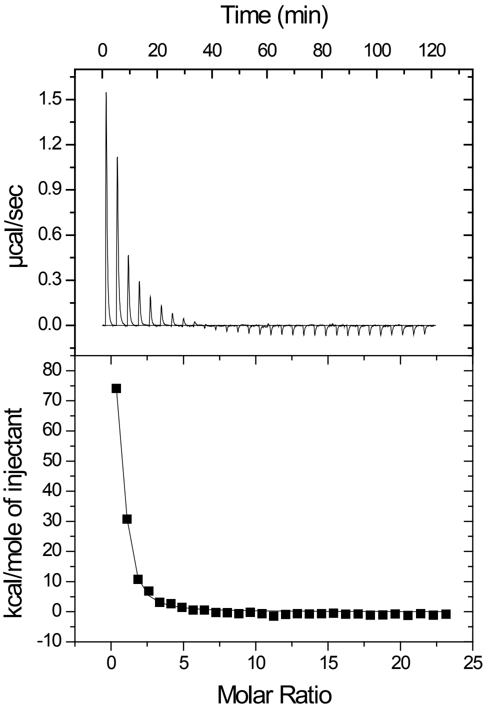
Time (min)
µcal/sec
kcal/mole of injectant
Molar Ratio

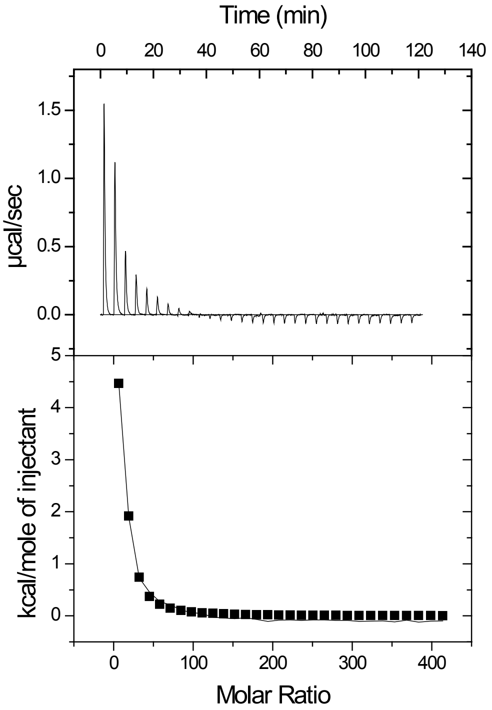
Time (min)
µcal/sec
kcal/mole of injectant
Molar Ratio

4. B20CH15 vs. Hemoglobin

N = 0.87 ± 2%   K = 3.99E6 ± 9% M^{-1}
ΔH = 81.6E ± 3% kcal M^{-1} ΔS = 0.34 kcal M^{-1} K^{-1}

Table S4: ITC titration data between B20CH15 and Hemoglobin in buffer.

C_{Hemo} [M]	$C_{B20CH15}$ [M]	Ratio $C_{B20CH15}/C_{Hemo}$	Heat exchange [μcal]
1.47E-06	5.72E-07	0.39	48.37
1.47E-06	1.71E-06	1.16	46.28
1.46E-06	2.84E-06	1.95	13.15
1.44E-06	3.97E-06	2.75	5.99
1.43E-06	5.08E-06	3.55	3.90
1.43E-06	6.19E-06	4.33	0.39
1.42E-06	7.28E-06	5.14	1.69
1.4E-06	8.38E-06	5.97	0.81
1.39E-06	9.46E-06	6.80	0.14
1.39E-06	1.05E-05	7.57	-2.71
1.38E-06	1.16E-05	8.42	-0.41
1.37E-06	1.27E-05	9.27	-0.45
1.35E-06	1.37E-05	10.14	-0.70
1.35E-06	1.48E-05	10.91	-0.70
1.34E-06	1.58E-05	11.78	-1.08
1.33E-06	1.68E-05	12.68	-1.00
1.31E-06	1.78E-05	13.58	-1.16
1.31E-06	1.88E-05	14.35	-1.09
1.3E-06	1.98E-05	15.26	-1.45
1.29E-06	2.08E-05	16.14	-1.47
1.28E-06	2.18E-05	17.03	-1.54
1.27E-06	2.28E-05	17.92	-1.66
1.26E-06	2.38E-05	18.81	-1.81
1.25E-06	2.47E-05	19.71	-2.91
1.25E-06	2.57E-05	20.62	-1.49
1.24E-06	2.66E-05	21.52	-1.74
1.23E-06	2.76E-05	22.44	-1.59
1.22E-06	2.85E-05	23.36	-1.92
1.21E-06	2.94E-05	24.29	-1.82
1.2E-06	3.00E-05	24.95	-1.88

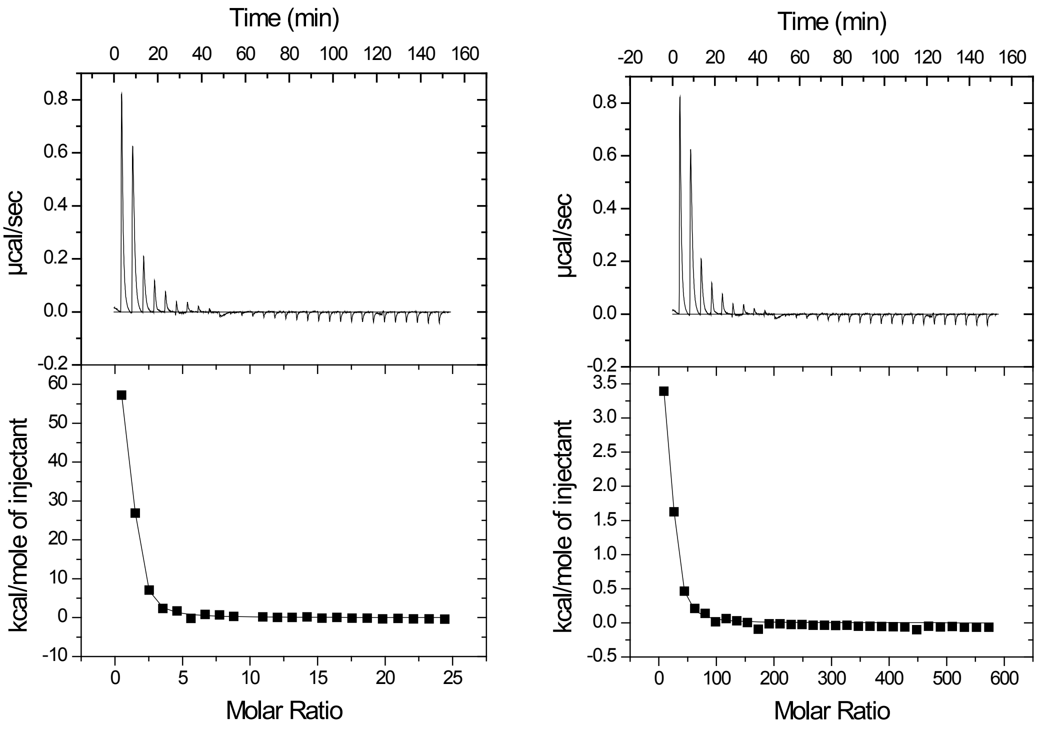
Time (min)
µcal/sec
kcal/mole of injectant
Molar Ratio
Time (min)
µcal/sec
kcal/mole of injectant
Molar Ratio

5. T20CH15 vs. Histone

Sequential binding two sites
$K_1 = 3.05E9 \pm 43\%\ M^{-1}$ $\Delta H_1 = 34.3 \pm 5\%$ kcal M^{-1} $\Delta S_1 = 0.161$ kcal M^{-1} K^{-1}
$K_2 = 2.51E5 \pm 9\%\ M^{-1}$ $\Delta H_2 = 158 \pm 1\%$ kcal M^{-1} $\Delta S_2 = 0.557$ kcal M^{-1} K^{-1}

Table S5: Dilution effect of T20CH15 in buffer.

Added volume μL	$C_{T20CH15}$ [M]	Heat exchange [μcal]
5	3.65E-07	-1.21
10	1.09E-06	8.54
10	1.81E-06	9.33
10	2.53E-06	9.74
10	3.24E-06	10.84
10	3.95E-06	10.42
10	4.65E-06	10.69
10	5.34E-06	10.93
10	6.03E-06	11.06
10	6.72E-06	10.52
10	7.40E-06	9.77
10	8.08E-06	10.12
10	8.75E-06	9.28
10	9.41E-06	9.65
10	1.01E-05	9.36
10	1.07E-05	8.43
10	1.14E-05	8.66
10	1.20E-05	9.22
10	1.27E-05	8.49
10	1.33E-05	8.09
10	1.39E-05	8.28
10	1.45E-05	8.13
10	1.52E-05	7.68
10	1.58E-05	7.92
10	1.64E-05	7.51
10	1.70E-05	7.19
10	1.76E-05	6.83
10	1.82E-05	7.01
10	1.88E-05	6.42
10	1.94E-05	7.06

ITC titration data between T20CH15 and Histone in buffer

$C_{Histone}$ [M]	$C_{T20CH15}$ [M]	Ratio $C_{T20CH15}/C_{Histone}$	Heat exchange [µcal]
3.87E-06	1.48E-06	0.38	53.92
3.84E-06	2.46E-06	0.64	58.63
3.81E-06	3.43E-06	0.90	67.92
3.79E-06	4.4E-06	1.16	101.46
3.76E-06	5.36E-06	1.43	116.79
3.74E-06	6.31E-06	1.69	94.28
3.71E-06	7.26E-06	1.96	73.29
3.68E-06	8.19E-06	2.23	62.44
3.66E-06	9.12E-06	2.49	55.75
3.63E-06	1.01E-05	2.77	49.58
3.61E-06	1.1E-05	3.04	45.19
3.58E-06	1.19E-05	3.32	41.05
3.56E-06	1.28E-05	3.59	38.10
3.53E-06	1.37E-05	3.87	34.44
3.51E-06	1.46E-05	4.15	33.51
3.48E-06	1.54E-05	4.44	30.31
3.46E-06	1.63E-05	4.72	28.83
3.43E-06	1.72E-05	5.01	25.06
3.41E-06	1.81E-05	5.29	25.12
3.38E-06	1.89E-05	5.59	24.04
3.36E-06	1.98E-05	5.88	21.12
3.34E-06	2.06E-05	6.16	19.03
3.31E-06	2.14E-05	6.47	21.39
3.29E-06	2.22E-05	6.76	17.15
3.27E-06	2.31E-05	7.05	16.18
3.24E-06	2.39E-05	7.37	16.35
3.22E-06	2.47E-05	7.66	14.50
3.2E-06	2.55E-05	7.96	14.39
3.17E-06	2.63E-05	8.29	12.43

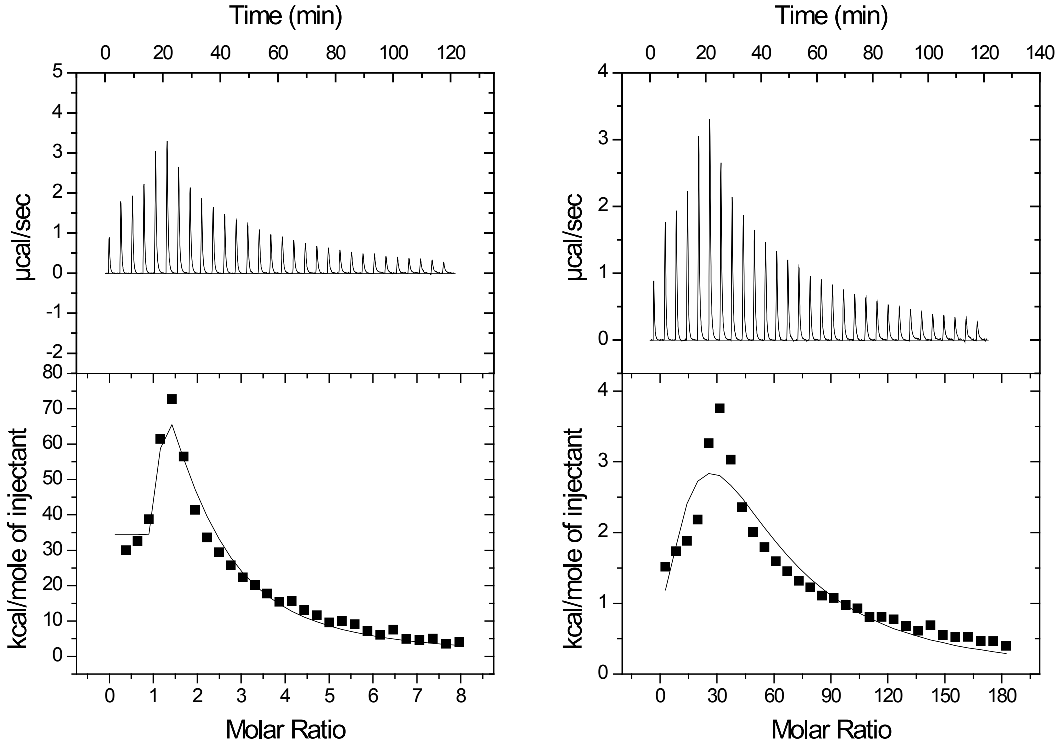
Time (min)
µcal/sec
kcal/mole of injectant
Molar Ratio
Time (min)
µcal/sec
kcal/mole of injectant
Molar Ratio

6. T20CH15 vs. Lysozyme

Sequential binding two sites
$K_1 = 7.69E5 \pm 22\%\ M^{-1}$ $\Delta H_1 = 69.4 \pm 7\%$ kcal M^{-1} $\Delta S_1 = 0.260$ kcal $M^{-1}\ K^{-1}$
$K_2 = 3.32E03 \pm 29\%\ M^{-1}$ $\Delta H_2 = -391 \pm 91\%$ kcal M^{-1} $\Delta S_2 = -1.30$ kcal $M^{-1}\ K^{-1}$

Table S6: ITC titration data between T20CH15 and Lysozyme in buffer.

$C_{Lysozyme}$ [M]	$C_{T20CH15}$ [M]	Ratio $C_{T20CH15}/C_{Lysozyme}$	Heat exchange [µcal]
3.39E-06	2.55E-06	0.75	57.36
3.37E-06	3.55E-06	1.05	55.61
3.34E-06	4.55E-06	1.36	43.29
3.32E-06	5.54E-06	1.67	30.70
3.30E-06	6.53E-06	1.98	23.74
3.27E-06	7.50E-06	2.29	19.62
3.25E-06	8.47E-06	2.61	17.02
3.23E-06	9.43E-06	2.92	15.79
3.20E-06	1.04E-05	3.25	13.73
3.18E-06	1.13E-05	3.57	13.81
3.16E-06	1.23E-05	3.89	12.72
3.14E-06	1.32E-05	4.21	12.29
3.12E-06	1.41E-05	4.53	11.41
3.09E-06	1.51E-05	4.87	9.64
3.07E-06	1.60E-05	5.20	10.79
3.05E-06	1.69E-05	5.53	10.03
3.03E-06	1.78E-05	5.86	9.25
3.01E-06	1.87E-05	6.20	8.18
2.99E-06	1.95E-05	6.54	8.12
2.96E-06	2.04E-05	6.90	7.68
2.94E-06	2.13E-05	7.24	7.50
2.92E-06	2.22E-05	7.59	6.80
2.90E-06	2.30E-05	7.93	6.37
2.88E-06	2.39E-05	8.28	7.42
2.86E-06	2.47E-05	8.63	1.92
2.84E-06	2.55E-05	8.99	6.91
2.82E-06	2.63E-05	9.34	5.72
2.80E-06	2.72E-05	9.70	4.87

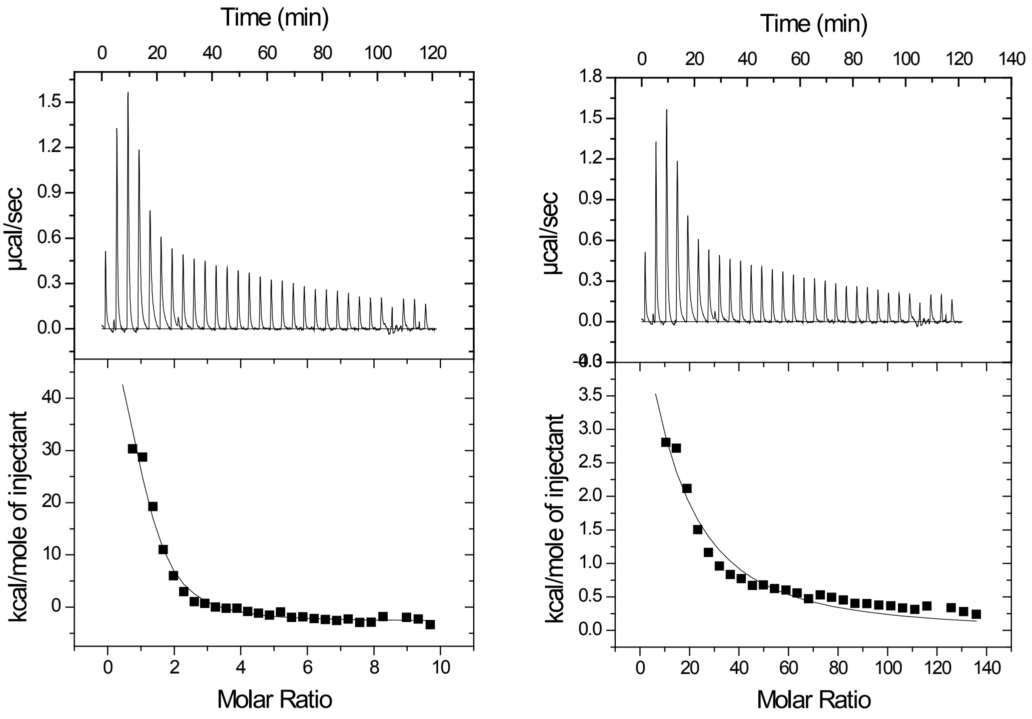
Time (min)
µcal/sec
kcal/mole of injectant
Molar Ratio
Time (min)
µcal/sec
kcal/mole of injectant
Molar Ratio

7. T20CH15 vs. Proteinase K

Sequential binding two sites
$K_1 = 4.44E5 \pm 27\%\ M^{-1}$ $\Delta H_1 = 36.6 \pm 12\%$ kcal M^{-1} $\Delta S_1 = 0.148$ kcal $M^{-1} K^{-1}$
$K_2 = 3.43E03 \pm 81\%\ M^{-1}$ $\Delta H_2 = -600 \pm 17\%$ kcal M^{-1} $\Delta S_2 = -1.99$ kcal $M^{-1} K^{-1}$

Table S7: ITC titration data between T20CH15 and Proteinase K in buffer.

$C_{Proteinase\ K}$ [M]	$C_{T20CH15}$ [M]	Ratio $C_{T20CH15}/C_{Pro.K}$	Heat exchange [µcal]
1.96E-06	1.43E-06	0.73	27.72
1.95E-06	2.37E-06	1.22	23.25
1.93E-06	3.31E-06	1.72	20.17
1.92E-06	4.24E-06	2.21	16.67
1.90E-06	5.17E-06	2.72	15.41
1.89E-06	6.09E-06	3.22	14.03
1.88E-06	7.00E-06	3.72	12.28
1.87E-06	7.90E-06	4.22	12.54
1.85E-06	8.80E-06	4.76	12.71
1.84E-06	9.69E-06	5.27	11.39
1.83E-06	1.06E-05	5.78	11.17
1.81E-06	1.15E-05	6.33	10.99
1.80E-06	1.23E-05	6.84	10.31
1.79E-06	1.32E-05	7.37	9.42
1.78E-06	1.41E-05	7.89	9.90
1.76E-06	1.49E-05	8.47	9.46
1.75E-06	1.57E-05	8.99	7.80
1.74E-06	1.66E-05	9.53	8.64
1.73E-06	1.74E-05	10.06	7.22
1.71E-06	1.82E-05	10.66	7.05
1.70E-06	1.91E-05	11.21	6.72
1.69E-06	1.99E-05	11.75	6.14
1.68E-06	2.07E-05	12.30	7.40
1.67E-06	2.15E-05	12.85	6.66
1.65E-06	2.23E-05	13.48	6.01
1.64E-06	2.30E-05	14.04	4.99
1.63E-06	2.38E-05	14.61	5.18
1.62E-06	2.46E-05	15.17	5.71
1.61E-06	2.53E-05	15.74	4.66

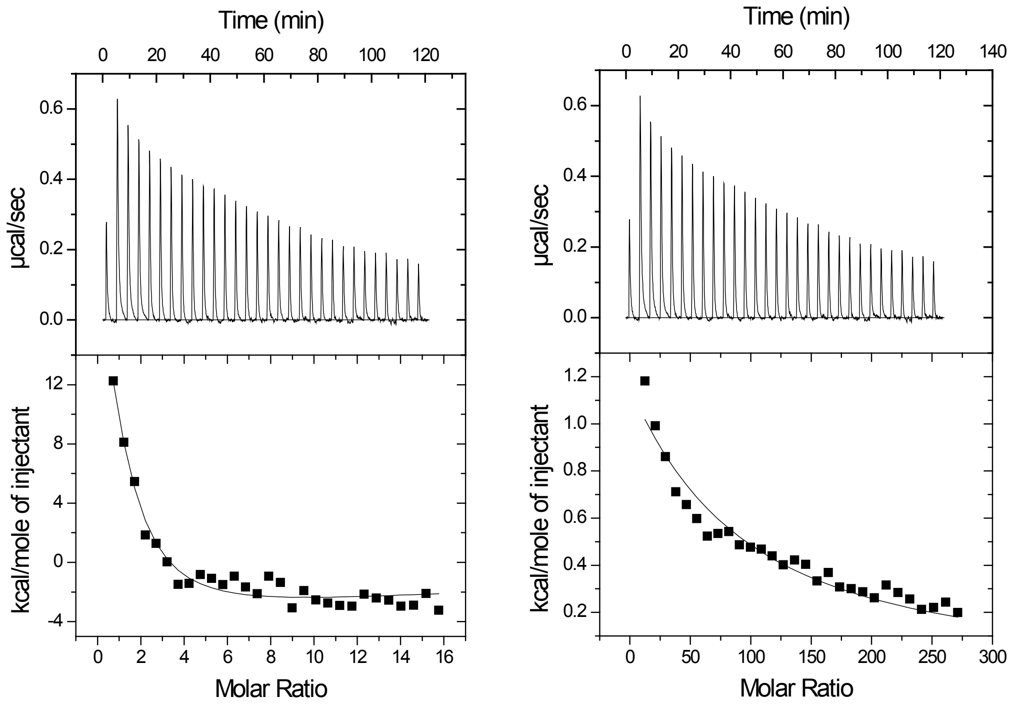

Time (min)
µcal/sec
kcal/mole of injectant
Molar Ratio
Time (min)
µcal/sec
kcal/mole of injectant
Molar Ratio

8. T20CH15 vs. BSA

Sequential binding two sites
$K_1 = 1.65E4 \pm 33\%\ M^{-1}$ $\Delta H_1 = 228 \pm 26\%$ kcal M^{-1} $\Delta S_1 = 0.783$ kcal $M^{-1}\ K^{-1}$
$K_2 = 9.44E03 \pm 19\%\ M^{-1}$ $\Delta H_2 = -246 \pm 87\%$ kcal M^{-1} $\Delta S_2 = -0.797$ kcal $M^{-1}\ K^{-1}$

Table S8: ITC titration data between T20CH15 and BSA in buffer.

C_{BSA} [M]	$C_{T20CH15}$ [M]	Ratio $C_{T20CH15}/C_{BSA}$	Heat exchange [µcal]
1.37E-05	4.17E-07	0.03	18.39
1.36E-05	1.25E-06	0.09	53.50
1.35E-05	2.07E-06	0.15	37.06
1.34E-05	2.89E-06	0.22	29.20
1.33E-05	3.70E-06	0.28	24.47
1.32E-05	4.51E-06	0.34	20.86
1.31E-05	5.31E-06	0.41	18.90
1.30E-05	6.10E-06	0.47	16.30
1.29E-05	6.89E-06	0.53	15.13
1.28E-05	7.68E-06	0.60	13.75
1.27E-05	8.45E-06	0.66	12.59
1.26E-05	9.22E-06	0.73	11.48
1.26E-05	9.99E-06	0.80	10.67
1.25E-05	1.08E-05	0.86	8.79
1.24E-05	1.15E-05	0.93	8.71
1.23E-05	1.23E-05	1.00	8.62
1.22E-05	1.30E-05	1.06	7.60
1.21E-05	1.37E-05	1.13	7.73
1.20E-05	1.45E-05	1.20	6.65
1.19E-05	1.52E-05	1.27	7.14
1.19E-05	1.59E-05	1.34	6.56
1.18E-05	1.66E-05	1.41	7.08
1.17E-05	1.73E-05	1.48	6.52
1.16E-05	1.80E-05	1.55	5.58
1.15E-05	1.87E-05	1.62	6.22
1.14E-05	1.94E-05	1.70	3.67
1.14E-05	2.01E-05	1.77	6.02
1.13E-05	2.08E-05	1.84	5.61
1.12E-05	2.14E-05	1.91	4.44
1.11E-05	2.21E-05	1.99	3.90

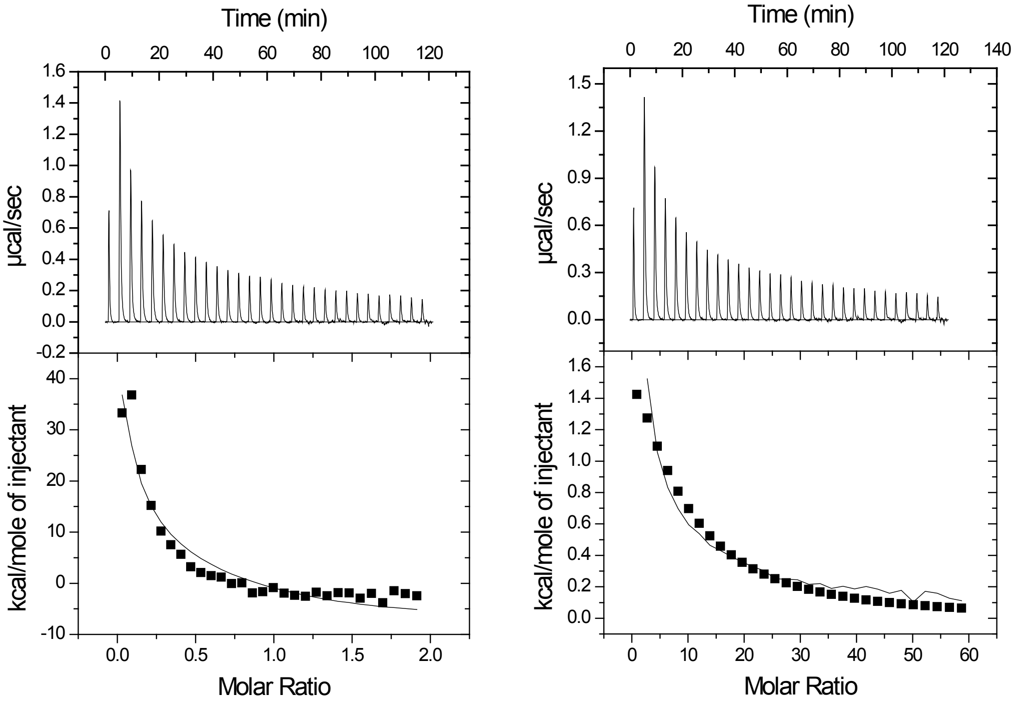
Time (min)
µcal/sec
kcal/mole of injectant
Molar Ratio
Time (min)
µcal/sec
kcal/mole of injectant
Molar Ratio

9. T20CH15 vs. Hemoglobin

N = 2.66 ± 3%   K = 4.39E6 ± 19% M^{-1}
ΔH = 5.17E4 ± 3% kcal M^{-1} ΔS = 0.24 kcal M^{-1} K^{-1}

Table S9: ITC titration data between T20CH15 and Hemoglobin in buffer.

C_{Hemo} [M]	$C_{T20CH15}$ [M]	Ratio $C_{T20CH15}/C_{Hemo}$	Heat exchange [µcal]
1.13E-06	4.53E-07	0.40	74.36
1.13E-06	1.35E-06	1.19	60.16
1.12E-06	2.25E-06	2.01	45.49
1.11E-06	3.14E-06	2.83	33.48
1.10E-06	4.02E-06	3.65	22.07
1.10E-06	4.90E-06	4.45	17.33
1.09E-06	5.77E-06	5.29	15.57
1.08E-06	6.63E-06	6.14	14.38
1.07E-06	7.49E-06	7.00	13.57
1.07E-06	8.34E-06	7.79	12.72
1.06E-06	9.18E-06	8.66	12.28
1.05E-06	1.00E-05	9.54	10.64
1.04E-06	1.09E-05	10.43	11.40
1.04E-06	1.17E-05	11.22	10.63
1.03E-06	1.25E-05	12.13	10.78
1.02E-06	1.33E-05	13.04	10.25
1.01E-06	1.41E-05	13.97	9.24
1.01E-06	1.49E-05	14.76	9.32
1.00E-06	1.57E-05	15.70	9.11
9.93E-07	1.65E-05	16.61	8.85
9.86E-07	1.73E-05	17.52	8.58
9.79E-07	1.80E-05	18.43	8.41
9.72E-07	1.88E-05	19.35	8.35

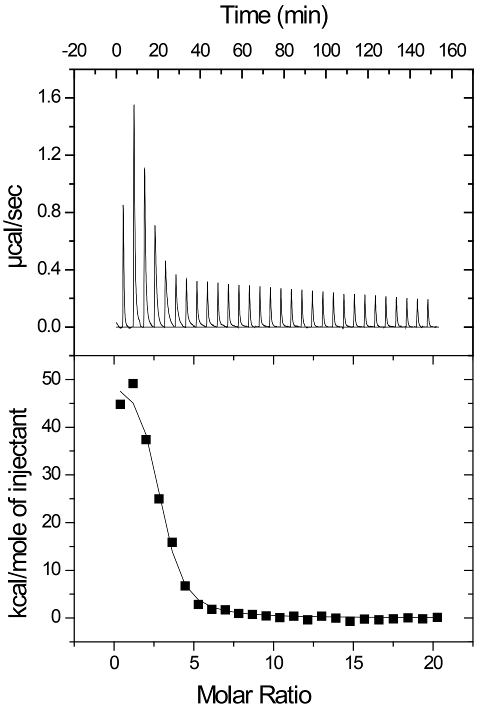
Time (min)
-20 0 20 40 60 80 100 120 140 160
µcal/sec
1.6
1.2
0.8
0.4
0.0
kcal/mole of injectant
50
40
30
20
10
0
0 5 10 15 20
Molar Ratio

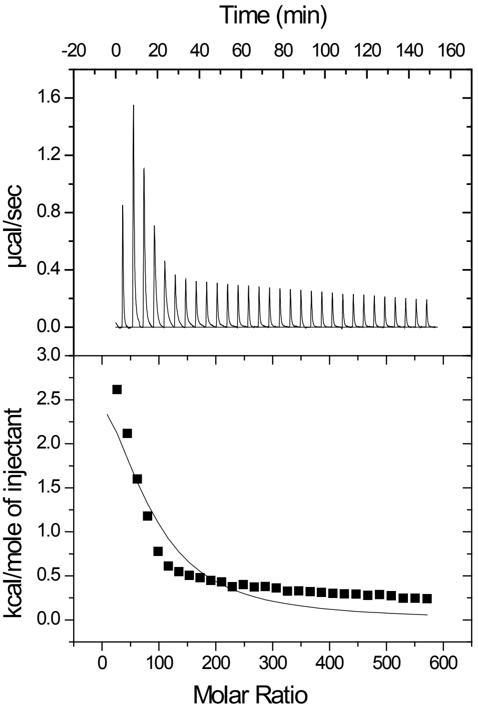
Time (min)
-20 0 20 40 60 80 100 120 140 160
µcal/sec
1.6
1.2
0.8
0.4
0.0
3.0
2.5
2.0
1.5
1.0
0.5
0.0
kcal/mole of injectant
0 100 200 300 400 500 600
Molar Ratio

10. S20CH15 vs. Cytochrome C

N= 7.31±51% K= 3.40E4 ± 28% M^{-1}
ΔH = 147 ± 68% kcal M^{-1} ΔS = 0.512 kcal M^{-1} K^{-1}

Table S10: ITC titration data between S20CH15 and Cytochrome C in buffer.

C_{Cyto} [M]	$C_{S20CH15}$ [M]	Ratio $C_{S20CH15}/C_{Cyto}$	Heat exchange [µcal]
1.39E-06	1.39E-06	1.00	47.96
1.38E-06	2.31E-06	1.67	47.54
1.37E-06	3.23E-06	2.36	46.27
1.36E-06	4.13E-06	3.04	43.37
1.35E-06	5.03E-06	3.73	40.62
1.34E-06	5.93E-06	4.43	37.74
1.33E-06	6.81E-06	5.12	35.69
1.32E-06	7.69E-06	5.83	33.88
1.31E-06	8.57E-06	6.54	31.76
1.30E-06	9.44E-06	7.26	30.58
1.29E-06	1.03E-05	7.98	29.18
1.28E-06	1.12E-05	8.71	28.09
1.27E-06	1.20E-05	9.45	26.31
1.27E-06	1.28E-05	10.11	24.88
1.26E-06	1.37E-05	10.85	24.55
1.25E-06	1.45E-05	11.60	23.45
1.24E-06	1.53E-05	12.35	22.75
1.23E-06	1.61E-05	13.12	21.77
1.22E-06	1.70E-05	13.89	21.16
1.21E-06	1.78E-05	14.67	21.00
1.20E-06	1.85E-05	15.45	19.43
1.20E-06	1.93E-05	16.11	19.73
1.19E-06	2.01E-05	16.90	18.97
1.18E-06	2.09E-05	17.70	18.47
1.17E-06	2.17E-05	18.51	18.01
1.16E-06	2.24E-05	19.33	17.92
1.15E-06	2.32E-05	20.16	16.41
1.15E-06	2.39E-05	20.81	15.98
1.14E-06	2.47E-05	21.64	16.42

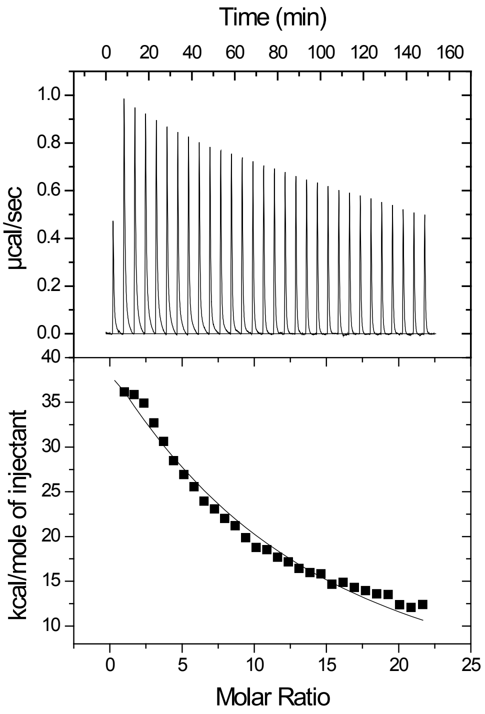
Time (min)
µcal/sec
kcal/mole of injectant
Molar Ratio

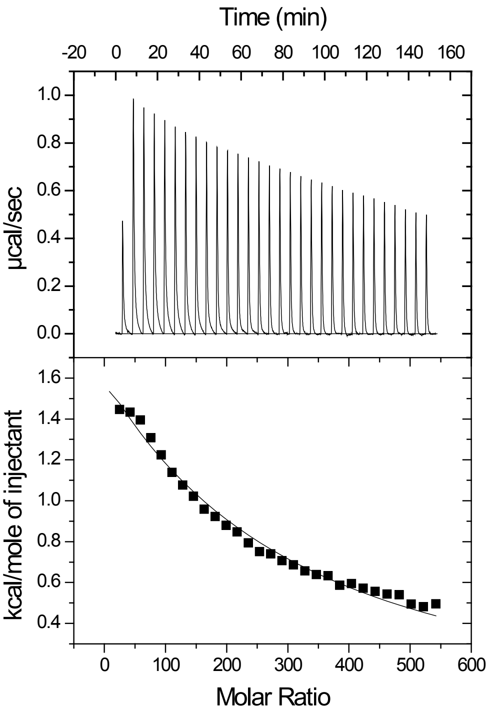
Time (min)
µcal/sec
kcal/mole of injectant
Molar Ratio

11. T20 vs. Hemoglobin

N = 1E−04 ± 38%   K = 2.02E4 ± 79% M^{-1}
ΔH = 846 ± 37% kcal M^{-1} ΔS = 2.86 kcal M^{-1} K^{-1}

Table S11: ITC titration data between T20 and Hemoglobin in buffer.

C_{Hemo} [M]	C_{T20} [M]	Ratio C_{T20}/C_{Hemo}	Heat exchange [µcal]
9.90E-04	1.05E-05	0.01	12.46
9.83E-04	1.74E-05	0.02	13.46
9.76E-04	2.43E-05	0.02	7.07
9.69E-04	3.12E-05	0.03	5.95
9.62E-04	3.80E-05	0.04	3.61
9.55E-04	4.47E-05	0.05	2.29
9.49E-04	5.14E-05	0.05	2.44
9.42E-04	5.80E-05	0.06	2.54
9.35E-04	6.46E-05	0.07	2.74
9.29E-04	7.12E-05	0.08	2.21
9.22E-04	7.77E-05	0.08	3.47
9.16E-04	8.41E-05	0.09	3.30
9.09E-04	9.05E-05	0.10	2.66
9.03E-04	9.68E-05	0.11	2.34
8.97E-04	1.03E-04	0.12	1.70
8.90E-04	1.09E-04	0.12	3.66
8.84E-04	1.16E-04	0.13	1.52
8.78E-04	1.22E-04	0.14	2.35
8.72E-04	1.28E-04	0.15	0.86
8.65E-04	1.34E-04	0.15	0.79
8.59E-04	1.40E-04	0.16	3.83
8.53E-04	1.46E-04	0.17	3.13
8.47E-04	1.52E-04	0.18	1.45
8.41E-04	1.58E-04	0.19	3.41
8.35E-04	1.63E-04	0.20	-0.05
8.29E-04	1.69E-04	0.20	0.18
8.24E-04	1.75E-04	0.21	-0.03
8.18E-04	1.80E-04	0.22	0.96
8.12E-04	1.86E-04	0.23	1.39

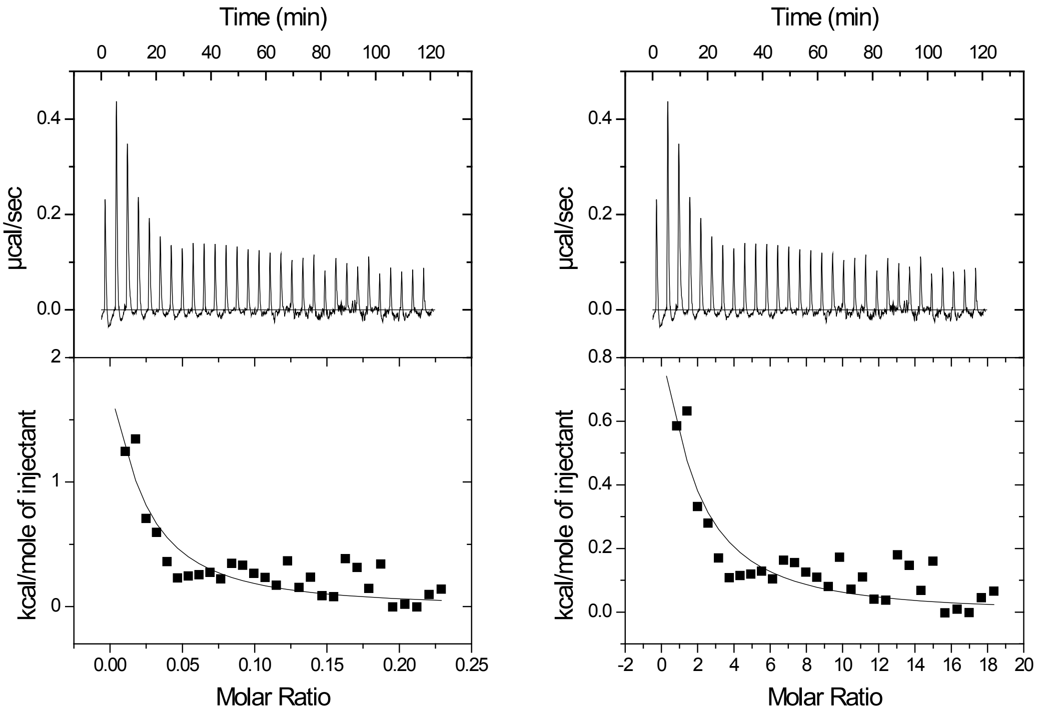
Time (min)
µcal/sec
kcal/mole of injectant
Molar Ratio
Time (min)
µcal/sec
kcal/mole of injectant
Molar Ratio

12. B20 vs Hemoglobin

N = 2.06 ± 16%   K = 6.88E5 ± 60% M^{-1}
ΔH = 11.8 ± 21% kcal M^{-1} ΔS = 0.066 kcal M^{-1} K^{-1}

Table S12: ITC titration data between B20 and Hemoglobin in buffer.

C_{Hemo} [M]	C_{B20} [M]	Ratio C_{B20}/C_{Hemo}	Heat exchange [µcal]
1.39E-06	1.39E-06	1.00	47.96
1.38E-06	2.31E-06	1.67	47.54
1.37E-06	3.23E-06	2.36	46.27
1.36E-06	4.13E-06	3.04	43.37
1.35E-06	5.03E-06	3.73	40.62
1.34E-06	5.93E-06	4.43	37.74
1.33E-06	6.81E-06	5.12	35.69
1.32E-06	7.69E-06	5.83	33.88
1.31E-06	8.57E-06	6.54	31.76
1.30E-06	9.44E-06	7.26	30.58
1.29E-06	1.03E-05	7.98	29.18
1.28E-06	1.12E-05	8.71	28.09
1.27E-06	1.20E-05	9.45	26.31
1.27E-06	1.28E-05	10.11	24.88
1.26E-06	1.37E-05	10.85	24.55
1.25E-06	1.45E-05	11.60	23.45
1.24E-06	1.53E-05	12.35	22.75
1.23E-06	1.61E-05	13.12	21.77
1.22E-06	1.70E-05	13.89	21.16
1.21E-06	1.78E-05	14.67	21.00
1.20E-06	1.85E-05	15.45	19.43
1.20E-06	1.93E-05	16.11	19.73
1.19E-06	2.01E-05	16.90	18.97
1.18E-06	2.09E-05	17.70	18.47
1.17E-06	2.17E-05	18.51	18.01
1.16E-06	2.24E-05	19.33	17.92
1.15E-06	2.32E-05	20.16	16.41
1.15E-06	2.39E-05	20.81	15.98
1.14E-06	2.47E-05	21.64	16.42

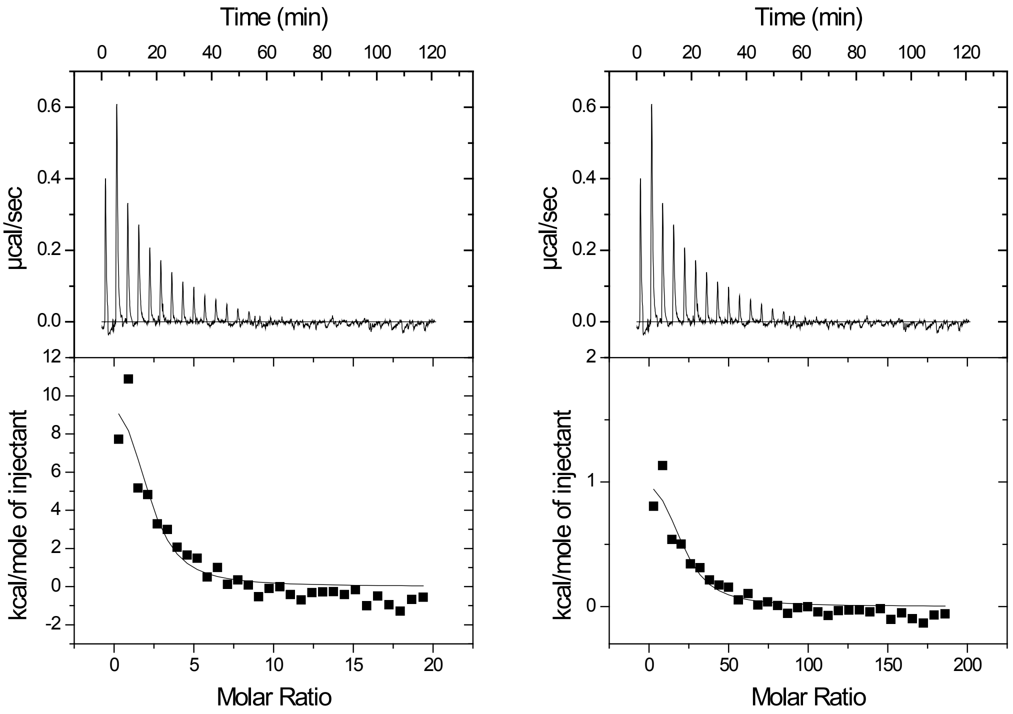

References:

[1] Renner, C.; Piehler, J.; Schrader, T., *J. Am. Chem. Soc.* **2006**, *128,* 620.
[2] J. Lai, D. Filla, R. Shea, *Macromolecules* **2002**, *35,* 6754.
[3] Lowe, A. B.; McCormick, C. L. *Prog. Polym. Sci*. **2007**, *32*, 283.
[4] Chong, Y. K.; Krstina, J.; Le, T. P. T.; Moad, G.; Postma, A.; Rizzardo, E.; Thang, S. H. *Macromolecules* **2003**, *36*, 2256.
[5] Y. Adewuyl and G. Carmichael, *Environ. Sci. Technol.* **1987**, *21,* 170.
[6] C. McCormick, A. Lowe, *Acc. Chem. Res*. **2004**, *37,* 312.
[7] Savitzky, A.; Golay, M. J. E. *Anal. Chem.* **1964**, *36,* 1627.
[8] Barak, P. *Anal. Chem.* **1995**, *67,* 2758.
[9] de Levie, R. In *Advanced Excel for Scientific Data Analysis,* Oxford University Press, Oxford 2004, p. 469.
